# Hepatic RACK1 deficiency protects against fulminant hepatitis through myeloid-derived suppressor cells

**DOI:** 10.7150/thno.65916

**Published:** 2022-02-14

**Authors:** Genyu Liu, Qingyang Wang, Lijiao Deng, Xiaofeng Huang, Guang Yang, Qianqian Cheng, Tingting Guo, Lu Guo, Chunxiao Niu, Xiqin Yang, Jie Dong, Jiyan Zhang

**Affiliations:** 1 Beijing Institute of Basic Medical Sciences, Beijing 100850, China.; 2 Chinese Institute for Brain Research, Beijing 102206, China.

**Keywords:** Fulminant Hepatitis, RACK1, HDAC1, Ubiquitination, MDSCs

## Abstract

Fulminant hepatitis (FH) is a life-threatening disease with partially understood pathogenesis. It has been demonstrated that myeloid-derived suppressor cells (MDSCs) are recruited into the liver during this process, and their augmented accumulation by various strategies protects against liver injury. However, the underlying mechanism(s) remain elusive. Receptor for activated C kinase 1 (RACK1), a multi-functional scaffold protein, is highly expressed in normal liver and has been implicated in liver physiology and diseases, but the *in vivo* role of hepatic RACK1 in FH remains unknown.

**Methods:** Survival curves and liver damage were monitored to investigate the *in vivo* role of hepatic RACK1 in FH. The liver microenvironment was explored by microarray-based transcriptome analysis, flow cytometry, immunoblotting, and immunohistochemistry. MDSCs were identified with phenotypic and functional characteristics. Functional antibodies were used to target MDSCs. Co-culture techniques were used to study the underlying mechanism(s) of protection. The interaction of RACK1 with histone deacetylase 1 (HDAC1) and the consequent effects on HDAC1 ubiquitination were analyzed. Ectopic expression of HDAC1 with recombinant adeno-associated virus serotype 8 was conducted to confirm the role of HDAC1 in the protective effects of hepatic RACK1 deficiency against FH. Post-translational modifications of RACK1 were also investigated during the induction of FH.

**Results:** Liver-specific RACK1 deficiency rendered mice resistant to FH. RACK1-deficient livers exhibited high basal levels of chemokine (C-X-C motif) ligand 1 (CXCL1) and S100 calcium-binding protein A9 (S100A9), associated with MDSC accumulation under steady-state conditions. Targeting MDSCs with an antibody against either Gr1 or DR5 abrogated the protective effects of liver-specific RACK1 deficiency. Accumulated MDSCs inhibited inflammatory cytokine production from macrophages and enhanced IκB kinase (IKK)/NF-κB pathway activation in hepatocytes. Further investigation revealed that RACK1 maintained HDAC1 protein level in hepatocytes by direct binding, thereby controlling histone H3K9 and H3K27 acetylation at the *Cxcl1* and *S100a9* promoters. Ectopic expression of HDAC1 in livers with RACK1 deficiency partially reversed the augmented *Cxcl1/S100a9* → MDSCs → IKK/NF-κB axis. During FH induction, RACK1 was phosphorylated at serine 110, enhancing its binding to ubiquitin-conjugating enzyme E2T and promoting its ubiquitination and degradation.

**Conclusion:** Liver-specific RACK1 deficiency protects against FH through accelerated HDAC1 degradation and the consequent CXCL1/S100A9 upregulation and MDSC accumulation.

## Introduction

Fulminant hepatitis (FH) is a life-threatening disease characterized by massive destruction of hepatocytes. Infections and/or hepatotoxic agents are the major causes of FH. The pathogenesis of FH is only partially understood. However, accumulating evidence suggests that immune cell activation and excessive cytokine production play an important role 1. For example, injection of mice with hepatotoxin D-Galactosamine (GalN) or immune cell-activating substance concanavalin A (Con A) leads to the inflammatory infiltration in hepatic parenchyma and the eventual acute liver failure 2, 3. These models demonstrated that myeloid-derived suppressor cells (MDSCs), a heterogeneous population of immature myeloid cells characterized by the surface co-expression of Gr-1 and CD11b and the inhibition of T-cell proliferation and/or IFN-γ production 4, are recruited into the liver. Increased accumulation of MDSCs by various strategies has been shown to protect against lipopolysaccharide (LPS)/GalN- or Con A-induced explosive hepatitis 5-8. Although interleukin-25 has been implicated 5, the mechanism(s) underlying the recruitment of MDSCs remain elusive. Furthermore, it is also unclear how MDSCs function to protect against liver injury.

Receptor for activated C kinase 1 (RACK1, initial gene name *Gnb2l1*) was originally identified for its ability to anchor activated form of protein kinase C (PKC) 9. RACK1 is highly expressed in normal liver and has been implicated in the liver physiology and diseases. For example, it has been reported that RACK1 expression in hepatic stellate cells is positively correlated with the clinical stage of liver fibrosis 10-12. RACK1 knockdown suppresses the expression of pro-fibrogenic factors *in vitro* and *in vivo* 10-12, and stimulation of cap-dependent translation was proposed as the key underlying mechanism 12. Elevated RACK1 expression has also been detected in hepatocellular carcinoma cells and has been demonstrated to promote oncogenic growth 13, 14. The underlying molecular mechanisms may be attributed to the preferential translation of the potent factors involved in growth and survival and direct interaction with mitogen-activated protein kinase kinase 7 (MKK7), enhancing its activity 13, 14. The role of RACK1 in translation has also been implicated in the replication of hepatitis C and E viruses in human liver cell lines 15, 16. As for the hepatitis B virus, its core protein has been shown to disrupt the interaction between MKK7 and RACK1, thereby enhancing tumor necrosis factor-α (TNF-α)-induced apoptosis in HepG2 human hepatoma cells 17. We have reported that RACK1 antagonizes TNF-α-induced apoptosis in primary murine hepatocytes 18. In addition, we have discovered that in a mouse model of liver-specific RACK1 deficiency, RACK1 works as an adaptor essential for the assembly of the autophagy-initiation complex and thereby prevents lipid accumulation in the liver 19. It has been shown that viral infections and TNF-α-induced cell death synergize to promote the development and progression of FH whereas autophagy protects against LPS/GalN-induced liver injury and mortality in mice 20-23. Despite these findings, the *in vivo* role of RACK1 in FH remains unknown.

This study reports that liver-specific RACK1 deficiency renders mice resistant to FH. Mechanistically, RACK1 directly interacts with and stabilizes co-repressor histone deacetylase 1 (HDAC1). In the absence of RACK1, HDAC1 is degraded. The consequently enhanced expression of chemokine (C-X-C motif) ligand 1 (CXCL1) and S100 calcium-binding protein A9 (S100A9) is associated with the accumulation of MDSCs under steady-state conditions. The accumulated MDSCs inhibit the production of inflammatory cytokines from macrophages and enhance the activation of the IκB kinase (IKK)/NF-κB pathway in hepatocytes. Consequently, liver injury is prevented.

## Materials and Methods

### Mice

*Rack1*^F/F^ and *Rack1*^Δhep^ mice on the C57BL/6 background, reported previously 19, were maintained under specific pathogen-free conditions. This study was approved by local Ethics Committee. All experiments were performed in accordance with institutional guidelines for animal care.

Mixed sexes and littermate controls were used for LPS/GalN- and TNF-α/GalN-induced liver damage, because phenotypes did not show gender dependency. 12-week-old mice were intraperitoneally injected with 1000 mg/kg GalN (G1639, Sigma-Aldrich, St. Louis, MO, USA) together with 35 μg/kg LPS (L7011, Sigma-Aldrich) or 15 μg/kg murine TNF-α (315-01A, PeproTech, London, UK) dissolved in 200 µl phosphate-buffered saline (PBS). For Con A-induced liver damage, 12-week-old male mice were intravenously injected with 25 µg/g Con A (C2010, Sigma-Aldrich) dissolved in 200 µL pyrogen-free saline.

To deplete MDSCs, 12-week-old mice were randomly divided into two groups. The control group was intraperitoneally injected once a day with 10 mg/kg IgG2b isotype control antibody (BE0090, Bio X Cell, Lebanon, NH, USA) and the other group was given anti-mouse Gr1 (Ly6G/Ly6C) antibody (BE0075, Bio X Cell, Lebanon NH, USA) at the same dose for three consecutive days. The mice were injected with LPS/GalN 24 h after the last injection of the antibody, as stated above. As an alternative way to target MDSCs, an anti-mouse DR5 antibody (200 μg/mouse, BE0161, Bio X Cell) was intraperitoneally injected 24 h before LPS/GalN administration.

For ectopic HDAC1 expression, 8-week-old *Rack1*^Δhep^ mice were randomly divided into two groups. One group was injected with 5 × 10^11^ v.g. recombinant adeno-associated virus serotype 8 (AAV8) with CMV promoter-driven expression of GFP in 200 μL PBS via the caudal vein, while the other group was injected with the same dose of AAV8 expressing murine FLAG-tagged HDAC1 (AAV8-HDAC1, Vigenebio, Jinan, China). Four weeks after injection, the mice were used for further experiments.

### Histological assessment

Mice were euthanised. The livers were removed and fixed for 48 h in 4% paraformaldehyde, dehydrated, infiltrated with paraffin, and sectioned at 5 μm. Slides were stained with hematoxylin and eosin (H & E) for brightfield microscopy.

### Measurement of transaminases

Blood samples were centrifuged at 3000 rpm for 10 min and the sera were collected. Alanine aminotransferase and aspartate aminotransferase levels were then assayed with commercial kits (ab105134 and ab105135, Abcam, Cambridge, UK) according to the manufacturer's protocols.

### Isolation of murine primary hepatocytes and cell culture

Primary hepatocytes were obtained from mice using an improved *in situ* collagen perfusion technique 24. In brief, mice were perfused with 37 °C HBSS (Ca^2+^ and Mg^2+^ free, Biotopped Life Sciences, Beijing, China) containing 0.5 mM EGTA through the hepatic portal system for 3 min, followed by perfusion with Dulbecco's modified Eagle's medium (DMEM) containing 10 mM HEPES, 0.075% collagenase I (C0130, Sigma-Aldrich), 100 U/mL penicillin, and 100 μg/mL streptomycin for an additional 5 min. The livers were removed and teased gently in a petri dish to loosen cells. Cell suspensions were transferred into DMEM containing 12.5% liver digest medium for 15 min at 37 °C. The samples were filtered using a sterile 70 μm nylon mesh. Cell suspensions were centrifuged at 50 × g, 4 °C for 5 min. Pellets were gently suspended in 5 mL DMEM and overlaid on 5 mL 40% Percoll solution in DMEM and centrifuged at 400 × g (up 6, down 2), 4 °C for 10 min. Primary hepatocytes were harvested from the interphase and washed twice in pre-chilled DMEM. Cell lines were purchased from the Shanghai Institutes for Biological Sciences. All adherent cells were cultured in DMEM supplemented with 10% fetal bovine serum (FBS), 100 U/mL penicillin, and 100 μg/mL streptomycin and were maintained at 37 ºC with 5% carbon dioxide.

### Plasmids and small interfering RNAs (siRNAs)

Mammalian expression vectors encoding Myc-tagged murine HDAC1 and HA-tagged human Ube2T (MG53562-NM and HG124409-CY) were purchased from Sino Biological Inc. (Beijing, China). The mammalian expression vector encoding FLAG-tagged human RAB40C (CH816878) was obtained from Vigenebio (Jinan, Chian). Other mammalian or prokaryotic expression vectors used in this study have been described previously 13, 19. Murine RACK1 siRNA (GGGCAAGATCATTGTAGAT) and the non-targeting control siRNA were purchased from Shanghai GenePharma (Shanghai, China). Transfection was performed with Lipofectamine 2000 (11668019, Invitrogen, Carlsbad, CA, USA) according to the manufacturer's protocol.

### Quantitative real-time PCR

RNA was extracted by using TRIzol reagent (15596-026, Invitrogen). First-strand synthesis was performed using the HiFiscript gDNA Removal RT Master Mix system (CW2582M, Markham, ON, Canada). Quantitative real-time PCR was performed with Fast Start Essential DNA Green Master (43993900, Roche Diagnostics, Mannheim, Germany) in a LightCycler® 96 System (Roche Diagnostics). Following primers were used: mouse *Cxcl1*, 5'-ctgggattcacctcaagaacatc-3' (forward) and 5'-cagggtcaaggcaagcctc-3' (reverse); mouse *S100a9*, 5'-caccctgagcaagaaggaat-3' (forward) and 5'- tgtcatttatgagggcttcattt-3' (reverse); mouse *Rack1*, 5'-ctggctacctgaacacagtgact-3' (forward) and 5'-ctgctggtgctgataacttcttg-3' (reverse); mouse *Hdac1*, 5'-gttgctcgctgctggacttac-3' (forward) and 5'-atcttcatccccactctcttcg-3' (reverse); and mouse *β-actin*, 5'-taaggccaaccgtgaaaagat-3' (forward) and 5'-ggagagcatagccctcgtagat-3' (reverse). Reaction mixtures were kept for 5 min at 95 ºC, followed by 45 cycles of 15 s at 95 ºC, 15 s at 60 ºC, and 30 s at 72 ºC. The foldchange in *Cxcl1*, *S100a9*, *Rack1*, or *Hdac1* expression, normalized to *β-actin* and relative to the expression in control group, was calculated for each sample as 2^-ΔΔ*C*T^, here Δ*C*_T_ = (*C*_T, *Target*_- *C*_T,_ *_β-actin_*) whereas ΔΔ*C*_T_ = (Δ*C*_T, condition x_- Avg.Δ*C*_T, control_) 25, 26.

### Microarray analysis

RNA was labeled with Cy5 dyes (Amersham Pharmacia, Uppsala, Sweden) and hybridized to Mouse Whole Genome OneArray with Phalanx hybridization buffer using Phalanx Hybridization System (Phalanx Biotech, San Diego, CA, USA). Data were analyzed according to the manufacturer's protocol, deposited in NCBI's GEO database, and are accessible through GEO Series accession numbers (record GSE164992 with reviewer token cjwjockmftqbhgz).

### Immunoblotting (IB) and co-immuno precipitation (Co-IP)

Cells or liver tissue were homogenized in RIPA buffer (50 mM Tris-Cl, pH 7.5, 1% NP40, 0.35% deoxycholate, 150 mM NaCl, 1 mM EDTA, 1 mM EGTA, supplemented with the protease and phosphatase inhibitor cocktail) or IP lysis buffer (10 mM Tris-Cl, pH 7.5, 2 mM EDTA, 1% NP40, 150 mM NaCl, supplemented with the protease and phosphates inhibitor cocktail). Then, the lysates were subjected to IB or Co-IP as previously described 13, 19. Antibodies against phospho-signal transducer and activator of transcription 3 (P-STAT3, Tyr705, 9134), phospho-IKKα/β (P-IKKα/β, Ser176/180, 2697), phospho-extracellular signal-regulated kinase 1/2 (P-ERK1/2, Thr202/Tyr204, 4370), phospho-c-Jun N-terminal kinase 1/2 (P-JNK1/2, Thr183/Tyr185, 4671), phospho-Akt (P-Akt, Ser473, 4060), phospho-threonine (P-Thr, 9386), Akt (4691), PARP (9532), and CXCL1/CXCL2 (24376) were purchased from Cell Signaling Technology (Danvers, MA, USA). Antibodies against RACK1 (sc-10775, for IP), IκBα (sc-4094), Bcl-2 (sc-7382), and protein A/G-plus agarose were from Santa Cruz Biotechnology (Santa Cruz, CA, USA). The antibody against RACK1 (610178, for IB) was obtained from BD Bioscience (San Jose, CA, USA). MG132 (M8699) and antibodies against FLAG tag (M2, F1804) and phospho-serine (P-Ser, P3430) were acquired from Sigma-Aldrich. Antibodies against Myc tag (M192-3) and GFP (598) came from MBL International (Woburn, MA, USA). The antibody against p50 was from Abcam. Antibody against HDAC1 (10197-1-AP), β-actin (66009-1-Ig), S100A9 (26992-1-AP), ubiquitin (Ub, 10201-2-AP), mouse double minute 2 (MDM2, 19058-1-AP), HA tag (51064-2-AP), p65 (10745-1-AP), and HDAC2 (12922-3-AP) were purchased from Proteintech (Chicago, IL, USA).

### Glutathione S-transferase (GST) pull-down assays

GST and GST-RACK1 were expressed and purified with glutathione-Sepharose (GSH) beads (17-0756-01, Amersham Pharmacia), according to the manufacturer's instruction. Lysates of BNL CL.2 cells expressing Myc-HDAC1 were incubated with GST or GST-RACK1 bound to GSH beads, and the adsorbed proteins were analyzed by IB analysis.

### Immunohistochemistry (IHC)

IHC was performed using standard protocols with citrate buffer (pH 6.0) pretreatment. Briefly, formaldehyde-fixed and paraffin-embedded liver sections were incubated with primary antibodies at 4 °C overnight and then with horseradish peroxidase-conjugated secondary antibodies at 37°C for 30 min. The sections were finally incubated with diaminobenzidine and counterstained with hematoxylin for detection. The antibody against RACK1 (610178) was ordered from BD Bioscience. The antibodies against HDAC1 (10197-1-AP) and S100A9 (26992-1-AP) were acquired from Proteintech.

### Isolation of liver mononuclear cells and flow cytometry analysis

Liver samples were minced and pressed through a 70-μm cell strainer. Cell suspensions were washed once in RPMI-1640 medium and centrifuged at 2000 rpm, 4 °C for 5 min. The pellets were suspended with 40% Percoll solution in RPMI-1640 medium and overlaid on 70% Percoll solution in RPMI-1640 medium and centrifuged at 400 × g (up 6, down 0), 4 °C for 20 min. Hepatic mononuclear cells were obtained from the interphase. Single-cell suspensions were washed with FACS washing buffer (2% FBS, 0.1% NaN_3_ in PBS) once and incubated with fluorescence-conjugated antibodies against cell surface molecules for 30 min on ice in the presence of 2.4G2 monoclonal antibody to block FcγR binding. Isotype antibodies were included as negative controls. After washing with FACS buffer, cells were fixed with 1% (w/v) paraformaldehyde in PBS and preserved at 4 ºC. Flow cytometry was performed using a Becton Dickinson FACS Fortessa machine (East Rutherford, NJ, USA) and data were analyzed using the FlowJo version 10. CD45-APC (30-F11, 103112), CD11b-PE-Cy7 (M1/70, 101216), Gr1-PE (RB6-8C5, 108408), F4/80-FITC (BM8, 123108), Ly6C-BV605 (HK1.4, 128036), Ly6G-BV421 (1A8, 127628), DR5-PE (MD5-1, 119906), and CD3-PERCP (17A2, 100218) were purchased from BioLegend (San Diego, CA, USA).

### Transwell co-culture

Primary hepatocytes (1 × 10^5^) were resuspended in 500 μL DMEM medium with 10% FBS and seeded into the bottom chamber of each well. Liver mononuclear cells were stained with Gr1-PE in FACS washing buffer without NaN_3_ for 20 min at 4 ºC. Then cells were incubated with anti-PE microbeads (130-048-801, Miltenyi Biotech, Bergisch Gladbach, Germany) for 20 min at 4 ºC. Gr1^+^ cells were harvested using an MS column (130-042-201, Miltenyi Biotech). The purity of Gr1^+^ cells was > 90% as confirmed by flow cytometry. Gr1^+^ cells were added to the inside of a transwell chamber (0.4 µm pore size, 19418020, Corning) at the same number of primary hepatocytes in the presence or absence of 50 μg/mL paquinimod (HY-100442, MedChemExpress, Monmouth Junction, NJ, USA). Twenty-four h later, the primary hepatocytes were exposed to 10 ng/mL TNF-α for various periods of time and subsequently harvested for IB.

### Carboxyfluorescein succinmidyl ester (CFSE) dilution

CD11b^+^F4/80^-^Ly6G^hi^Ly6C^lo^ cells were sorted from the liver mononuclear cells of *Rack1*^Δhep^ mice before and 2 h after LPS/GalN challenge. CD4^+^ T cells in the spleen of *Rack1*^F/F^ mice were isolated using a CD4^+^ T cell isolation kit (130-104-454, Miltenyi Biotec, Bergisch Gladbach, Germany) according to the manufacturer's instruction. The purity of these subsets was around 95% as confirmed by flow cytometry. Purified CD4^+^ T cells were labeled with 1 μM CFSE (65-0850-84, eBioscience) at 37 ºC for 20 min. After thorough wash, CD4^+^ T cells were cultured with or without two-fold number of these myeloid cells in RPMI-1640 medium supplemented with 10% FBS, 100 U/mL penicillin, 100 μg/mL streptomycin, 2 mM L-glutamine, and 50 μM β-mercaptoethanol in the presence or absence of Dynabeads Mouse T-Activator CD3/CD28 (11456D, Gibco, New York, MA, USA) at 37 ºC for 72 h. T-cell proliferation was determined by CFSE dilution.

### Measurement of inflammatory cytokines

About 30 mg of the liver tissue was cut and homogenized in 10 volumes of PBS (pH7.5) using a pellet pestle cordless motor (SIGMA, Darmstadt, Germany). Samples were then centrifuged at 3000 rpm for 10 min and the supernatants were collected and stored at -80 ºC until use. CD3^+^ T cells sorted from the livers of *Rack1*^F/F^ mice were stimulated with 1 μg/mL phorbol myristate acetate (P8139, Sigma-Aldrich) and 1 μM ionomycin (I3909, Sigma-Aldrich) in the presence or absence of the same number of CD11b^+^F4/80^-^Ly6G^hi^Ly6C^lo^ myeloid cells sorted from the livers of *Rack1*^Δhep^ mice, and the supernatants were collected 4 h later. Bone marrow-derived macrophages from *Rack1*^F/F^ mice were stimulated with 100 ng/mL LPS in the presence or absence of the same number of CD11b^+^F4/80^-^Ly6G^hi^Ly6C^lo^ myeloid cells sorted from the livers of *Rack1*^Δhep^ mice, and the supernatants were collected 4 h later. TNF-α, IL-6, IL-17A, and IFN-γ levels in the supernatants were assayed with commercial kits 88-7324-88, 88-7064-88, 88-7314-88, and 88-7371-88 (eBioscience, San Diego, CA, USA), respectively, according to the manufacturer's protocols.

### Indirect immunofluorescence

Primary hepatocytes were cultured in sterile collagen-coated fluorescent dishes. Cells were fixed in 4% (w/v) paraformaldehyde in PBS for 30 min at room temperature and then permeabilized with 0.5% Triton X-100 in PBS for 15 min. Non-specific sites were blocked by incubation with 5% goat serum and 0.3% Triton X-100 in PBS for 1 h at room temperature. Samples were then incubated with an anti-RACK1 (610178, BD Bioscience) or anti-HDAC1 (10197-1-AP, Proteintech) antibody overnight at 4℃. After washing three times in PBS containing 0.05% Tween 20, cells were incubated with TRITC- or FITC-conjugated secondary antibodies for 45 min at room temperature. Cells were rewashed as stated above, mounted with DAPI (ZLI-9557, Origene, Rockville, MD, USA), and then observed by laser scanning confocal microscopy (RADIANCE 2100, Bio-Rad, Hercules, CA, USA).

### Chromatin immunoprecipitation (ChIP)

ChIP was performed with a SimpleChIP® Plus Sonication ChIP kit (56383, Cell Signaling Technology) according to the manufacturer's instruction. Briefly, the liver tissue was harvested and fixed for 10 min at room temperature with 1% formaldehyde. After shearing the genomic DNA by sonication, Protein G Magnetic Beads and a primary rabbit antibody or normal rabbit IgG were added to each sample, followed by incubation at 4 °C overnight with rotation. After reversal of protein-DNA cross-links, DNA was purified using DNA purification spin columns and subjected to quantitative PCR. Antibodies against HDAC1 (34589), acetyl-Histone H3K9 (9649), and acetyl-Histone H3K27 (8173) were ordered from Cell Signaling Technology.

### *In vivo* ubiquitination assay with IP

Cells or liver tissue were solubilized in modified lysis buffer (50 mM Tris-Cl, pH 7.4, 150 mM NaCl, 10% glycerol, 1 mM EDTA, 1 mM EGTA, 1% SDS, 1 mM Na_3_VO_4_, 1 mM DTT, and 10 mM NaF) supplemented with the protease inhibitor cocktail, as previously described 27. Lysates were incubated at 60 ºC for 10 min, followed by 10 times dilution with modified lysis buffer without SDS. After sonication, samples were incubated at 4 ºC for 1 h with rotation, followed by centrifugation (14 000 rpm) for 30 min at 4 °C. The protein concentration was determined by the Bradford assay, and appropriate amounts (0.5-1.5 mg) of protein were used for immunoprecipitation. Immunoprecipitated proteins were washed with washing buffer (50 mM Tris-Cl, pH 7.4, 500 mM NaCl, 10% glycerol, 1 mM EDTA, 1 mM EGTA, 0.1% SDS, 1 mM DTT, and 10 mM NaF) three times, boiled in SDS sample buffer, and separated on SDS-PAGE.

### Statistical analysis

Quantitative data are shown as mean ± standard deviations (SD) and were assessed by one-way ANOVA or Student's *t* test. Survival curves of two groups were compared using the log-rank (Mantel-Cox) test. Statistical calculations were performed using Prism 6. All statistical tests were two-sided, and *P* values of less than 0.05 were considered statistically significant. *p < 0.05; **p < 0.01; ***p < 0.001.

## Results

### Liver-specific RACK1 deficiency renders mice resistant to FH

**Figure 1:**
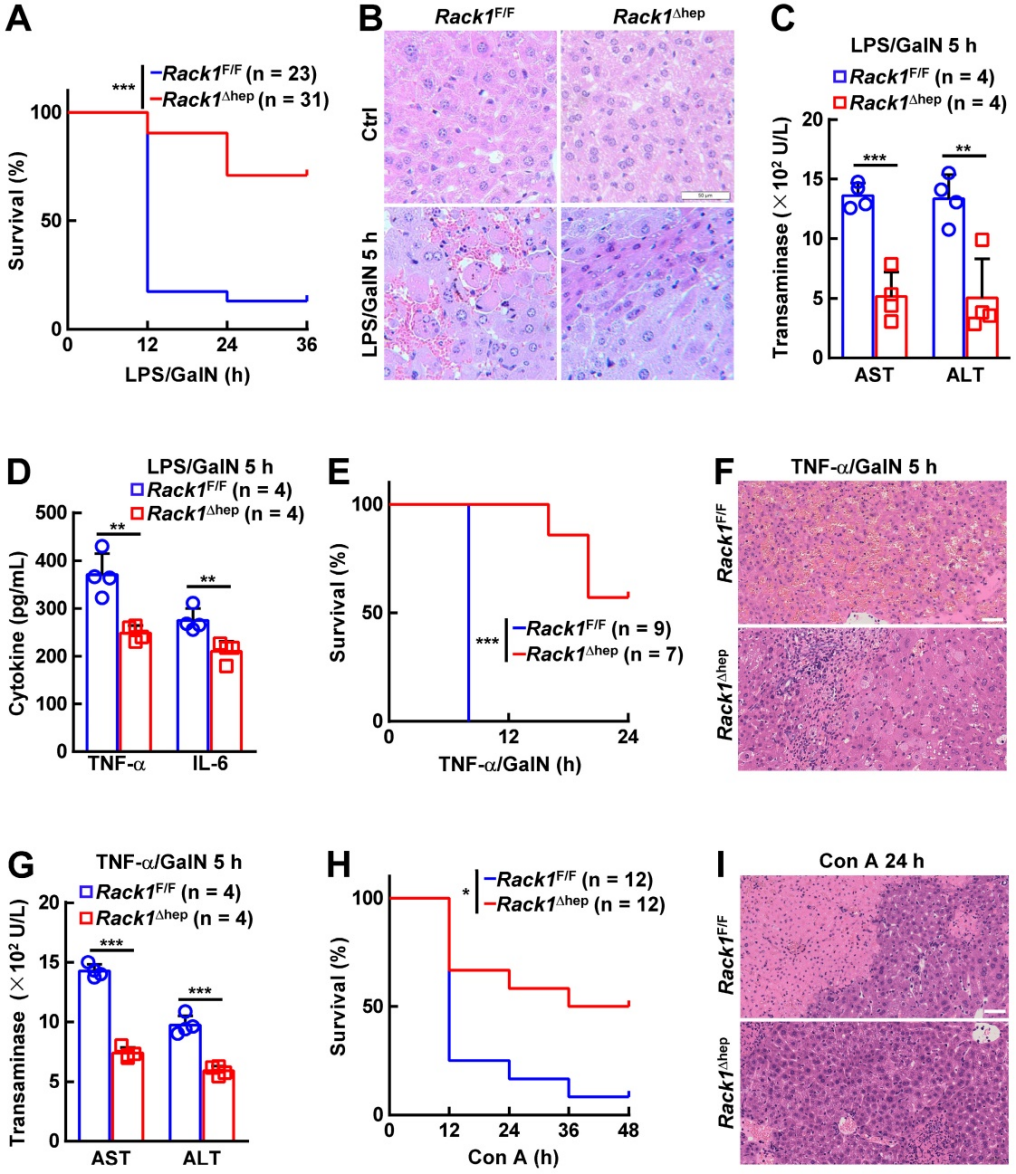
** Liver-specific RACK1 deficiency renders mice resistant to FH.** 12-week-old *Rack1*^Δhep^ mice and littermate controls were injected with LPS (35 μg/kg) and GalN (1000 mg/kg) intraperitoneally (mixed sex, **A-D**), TNF-α (15 µg/kg) and GalN (1000 mg/kg) intraperitoneally (mixed sex, **E-G**), or Con A (25 µg/g) intravenously (male only, **H, I**). Survival curves (**A, E, H**), representative histology of liver sections (**B**, **F**, **I**, scale bar, 50 µm), ALT and AST levels (**C, G**), and inflammatory factors in the liver tissue (**D**) are shown. U/L, units per liter. Error bars indicate mean ± SD. The n-value information is shown in the corresponding panel. *p < 0.05; **p < 0.01; ***p < 0.001; NS, not significant.

The *in vivo* role of RACK1 in FH was explored by administering LPS/GalN in 12-week-old *Rack1*^Δhep^ mice and littermate controls. Unexpectedly, only about 10% of *Rack1*^F/F^ mice versus more than 70% of *Rack1*^Δhep^ littermates survived over 36 h (Figure 1A). Histology revealed that LPS/GalN treatment for 5 h led to extensive necrosis in livers of *Rack1*^F/F^ mice but not in *Rack1*^Δhep^ mice (Figure 1B). Furthermore, serum levels of alanine aminotransferase (ALT) and aspartate transaminase (AST), markers of hepatocyte damage, were significantly higher in* Rack1*^F/F^ mice than in *Rack1*^Δhep^ littermates 5 h after LPS/GalN administration (Figure 1C). The decreased damage in RACK1-deficient livers was associated with reduced tissue levels of inflammatory factors TNF-α and IL-6 (Figure 1D). Similarly, liver-specific RACK1 deficiency led to better survival (Figure 1E), alleviated hepatocyte necrosis (Figure 1F), and lower levels of serum ALT and AST (Figure 1G) after TNF-α/GalN administration. The protective role of hepatic RACK1 deficiency was also observed after the administration of Con A (Figure 1H and 1I).

### Liver-specific RACK1 deficiency leads to MDSC accumulation

**Figure 2:**
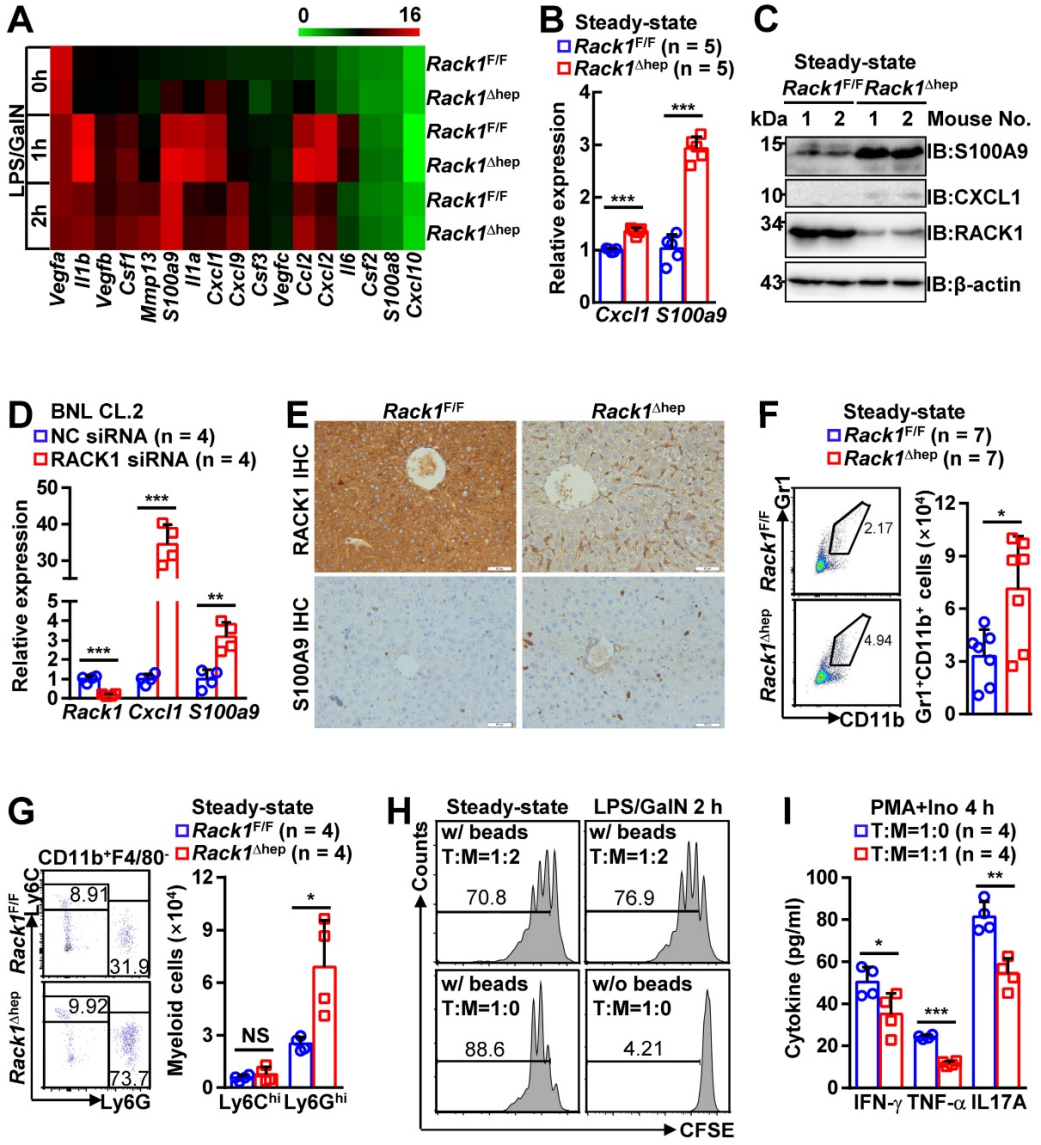
** Liver-specific RACK1 deficiency leads to MDSC accumulation under steady-state conditions.** (**A**) 12-week-old *Rack1*^Δhep^ mice and littermate controls were injected with LPS/GalN. After the indicated time periods, livers were isolated and subjected to microarray analysis. Genes involved in the recruitment and local expansion of MDSCs are shown in heatmaps. Mean values from two mice are shown. (**B, C, E**) Livers of 12-week-old *Rack1*^Δhep^ mice and littermate controls were subjected to quantitative RT-PCR (B), IB (C), and IHC (E, scale bar, 50 µm) to examine CXCL1 and S100A9 expression. (**D**) 48 h after transfection with siRNA against murine RACK1 or non-targeting control (NC) siRNA, BNL CL.2 cells were subjected to quantitative RT-PCR. (**F, G**) Liver mononuclear cells were purified from 12-week-old *Rack1*^Δhep^ mice and littermate controls, followed by flow cytometry analysis of Gr1^+^CD11b^+^ subset in CD45^+^ cells (F) or Ly6G^hi^Ly6C^lo^ and Ly6G^lo^Ly6C^hi^ subsets in CD45^+^CD11b^+^F4/80^-^ cells (G). Representative plots (*left*) and statistical data (*right*) are shown. (**H**) CD11b^+^F4/80^-^Ly6G^hi^Ly6C^lo^ myeloid cells were sorted from livers of *Rack1*^Δhep^ mice before and 2 h after LPS/GalN challenge. CFSE-labeled CD4^+^ T cells from spleens of *Rack1*^F/F^ mice were cultured with or without two-fold number of these myeloid cells in the presence (w/) or absence (w/o) of Dynabeads Mouse T-Activator CD3/CD28. Proliferation was assessed by flow-cytometric analysis of CFSE dilution 72 h later. (**I**) CD3^+^ T cells sorted from livers of *Rack1*^F/F^ mice were stimulated with 1 μg/mL phorbol myristate acetate (PMA) and 1 μM ionomycin (Ino) for 4 h in the presence or absence of the same number of CD11b^+^F4/80^-^Ly6G^hi^Ly6C^lo^ myeloid cells sorted from livers of *Rack1*^Δhep^ mice. IFN-γ, TNF-α, and IL-17A levels in the supernatants were then determined by ELISA. Error bars indicate mean ± SD. The n-value information is shown in the corresponding panel. *p < 0.05; **p < 0.01; ***p < 0.001; NS, not significant.

The role of liver-specific RACK1 deficiency in LPS/GalN-, TNF-α/GalN-, or Con A-induced FH was contrary to the finding that RACK1 knockdown aggravates TNF-α-induced apoptosis of cultured primary murine hepatocytes 18. This paradox suggested an altered microenvironment rather than a cell autonomous role. We performed a microarray-based transcriptome analysis of *Rack1*^F/F^ and *Rack1*^Δhep^ livers before and after LPS/GalN challenge. Unexpectedly, *Rack1*^F/F^ and *Rack1*^Δhep^ livers showed comparable levels of *Tnf*, *Il6*, and *Il1b* before and after LPS/GalN challenge, suggesting that reduced inflammatory cytokine levels in RACK1-deficient livers might result from post-transcriptional regulation. However, several genes involved in the recruitment and/or local expansion of MDSCs, especially *Cxcl1* and *S100a9* 28-30, were markedly upregulated in *Rack1*^Δhep^ livers even under steady-state conditions (Figure 2A). These findings were confirmed by quantitative RT-PCR (Figure 2B) and IB analysis (Figure 2C). The role of RACK1 in limiting CXCL1 and S100A9 expression was cell-autonomous since silencing of endogenous RACK1 expression by RACK1 siRNA in BNL CL.2 normal murine hepatocytes led to increased mRNA levels of *Cxcl1* and *S100a9* (Figure 2D). It has been reported that S100A9 was chemotactic for MDSCs, which, in turn, produced increased S100A9 31, 32. Indeed, steady-state *Rack1*^Δhep^ livers showed slightly elevated S100A9 expression in hepatocytes and more S100A9^hi^ infiltrated immune cells, as indicated by IHC (Figure 2E), suggesting MDSC accumulation. Therefore, liver mononuclear cells were isolated and subjected to flow cytometry analysis. As expected, *Rack1*^Δhep^ mice showed higher numbers of Gr1^+^CD11b^+^ cells in the liver than their littermate controls under steady-state conditions (Figure 2F).

MDSCs include two major subsets: polymorphonuclear-MDSCs (CD11b^+^Ly6G^hi^Ly6C^lo^) and monocytic-MDSCs (CD11b^+^Ly6G^lo^Ly6C^hi^) 4. Flow cytometry analysis revealed the accumulation of CD11b^+^F4/80^-^Ly6G^hi^Ly6C^lo^ cells, but not CD11b^+^F4/80^-^Ly6G^lo^Ly6C^hi^ cells, in steady-state *Rack1*^Δhep^ livers (Figure 2G). MDSCs are usually described under pathogenic conditions characterized by the inhibition of T-cell proliferation and/or IFN-γ production 4. To explore whether these accumulated myeloid cells possess immunosuppressive function under steady-state conditions, we sorted CD11b^+^F4/80^-^Ly6G^hi^Ly6C^lo^ cells from *Rack1*^Δhep^ livers before and after LPS/GalN challenge and mixed them with CFSE-labeled splenic CD4^+^ T cells. After co-culturing for 72 h in the presence or absence of antibodies against CD3 and CD28, flow cytometry analysis of CFSE dilution revealed that myeloid cells accumulated either under steady-state conditions or under disease conditions partially inhibited T-cell proliferation with similar efficiency (Figure 2H). We further analyzed whether the accumulated myeloid cells inhibited T-cell cytokine production. We found that phorbol myristate acetate- and ionomycin-induced IFN-γ, TNF-α, and IL-17A production in liver CD3^+^ T cells was significantly inhibited by CD11b^+^F4/80^-^Ly6G^hi^Ly6C^lo^ cells sorted from steady-state *Rack1*^Δhep^ livers (Figure 2I). Together, these data confirmed that liver-specific RACK1 deficiency resulted in MDSC accumulation in this organ under steady-state conditions.

### Targeting MDSCs abrogates protective effects of hepatic RACK1 deficiency

**Figure 3:**
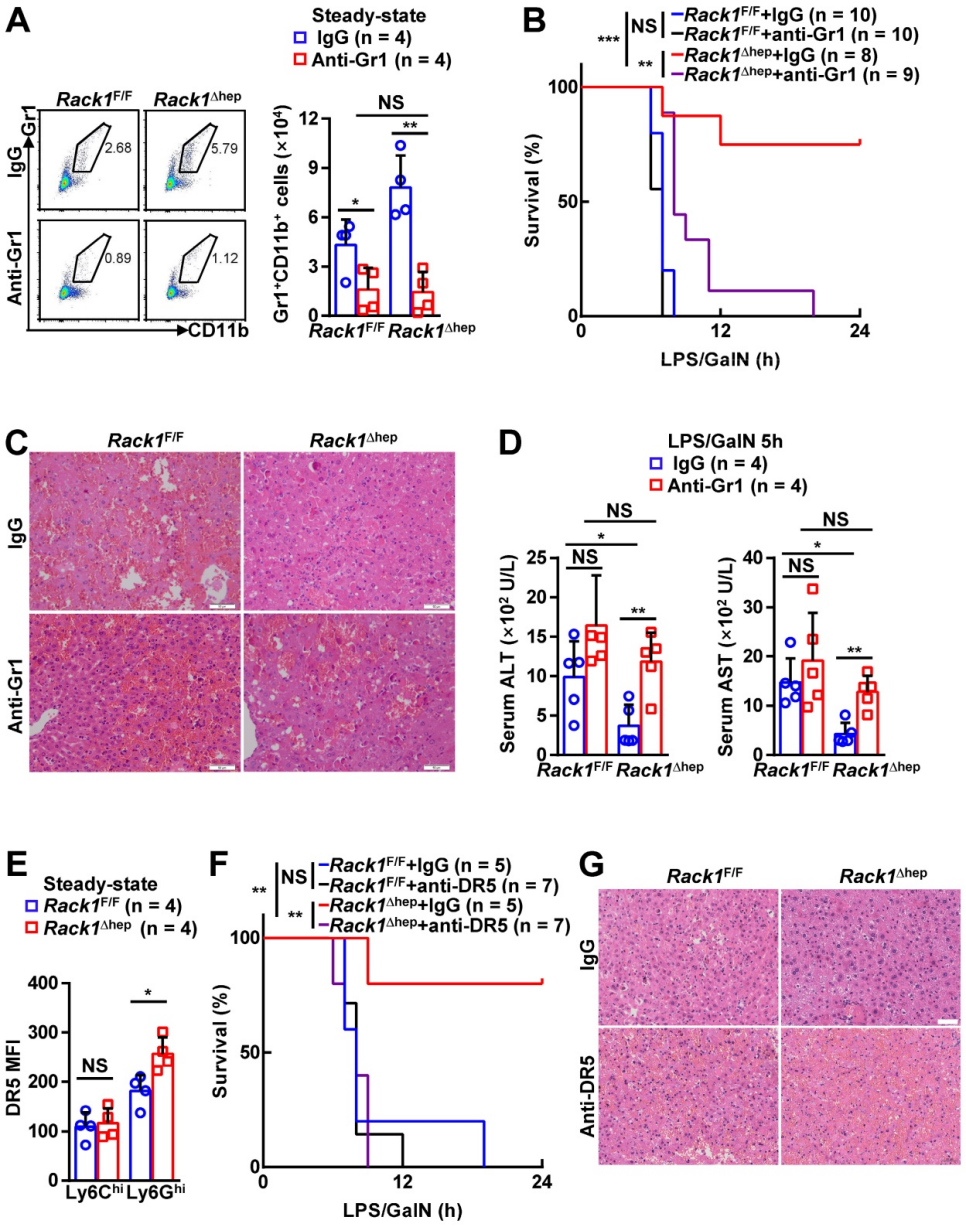
** Targeting MDSCs abrogates protective effects of hepatic RACK1 deficiency.** (**A**) 12-week-old *Rack1*^Δhep^ mice and littermate controls were intraperitoneally injected once a day with 10 mg/kg IgG2b isotype control antibody or anti-mouse Gr1 (Ly6G/Ly6C) antibody for three consecutive days. Then, liver mononuclear cells were purified and subjected to flow cytometry analysis of Gr1^+^CD11b^+^ subset in CD45^+^ cells. Representative plots (*left*) and statistical data (*right*) are shown. (**B-D**) After the depletion of MDSCs, mice were injected with LPS/GalN, and their survival was monitored (**B**). Representative histology of liver sections (**C**, scale bar, 50 µm) and serum levels of ALT and AST (**D**) 5 h after the injection are shown. (**E**) Liver mononuclear cells were purified from 12-week-old *Rack1*^Δhep^ mice and littermate controls, followed by flow cytometry analysis of DR5 mean fluorescence intensity (MFI) in Ly6G^hi^Ly6C^lo^ and Ly6G^lo^Ly6C^hi^ subsets of MDSCs. (**F, G**) 12-week-old *Rack1*^Δhep^ mice and littermate controls were intraperitoneally injected with IgG2b isotype control antibody or anti-mouse DR5 antibody at the 200 μg/mouse dose, followed by LPS/GalN injection 24 h later and their survival was monitored (**F**). Representative histology images of liver sections (**G**, scale bar, 50 µm) 5 h after LPS/GalN injection are shown. Error bars indicate mean ± SD. The n-value information is shown in the corresponding panel. *p < 0.05; **p < 0.01; ***p < 0.001; NS, not significant.

Locally accumulated Gr1^+^CD11b^+^ myeloid cells have been demonstrated to exert protective function against LPS/GalN- and Con A-induced liver damage 5-8. To explore whether the accumulation of MDSCs mediates the protective role of hepatic RACK1 deficiency, we deleted MDSCs by injection of a Gr1 (Ly6G/Ly6C) neutralizing antibody. Isolation of liver mononuclear cells followed by flow cytometry analysis confirmed the efficacy of the depleting antibody, compared with control antibody IgG2b (Figure 3A). After injecting the control antibody, *Rack1*^F/F^ mice died rapidly following LPS/GalN administration, whereas most *Rack1*^Δhep^ mice survived under the same conditions (Figure 3B), consistent with our data as mentioned earlier (Figure 1A). Anti-Gr1 antibody treatment resulted in rapid death of *Rack1*^Δhep^ mice (Figure 3B). Furthermore, histology analysis (Figure 3C) and measurement of serum transaminases (Figure 3D) showed that the protective effects of hepatic RACK1 deficiency against LPS/GalN-driven acute liver damage diminished after depletion of MDSCs. Notably, anti-Gr1 antibody treatment also showed a tendency to accelerate LPS/GalN-induced death (*p* = 0.0677) (Figure 3B) and augment LPS/GalN-induced elevation of serum ALT (*p* = 0.0981) (Figure 3D) in *Rack1*^F/F^ mice and was associated with aggravated hepatocyte necrosis (Figure 3C).

Since anti-Gr1 antibody treatment could also deplete neutrophils 33, a more specific antibody should be used to target MDSCs. Activation of endoplasmic reticulum stress is a common feature of MDSCs in both mice and humans, inducing the upregulation of DR5, a TRAIL receptor 34, 35. It has been reported that targeting DR5 could specifically induce MDSC apoptosis 35, 36. Elevated DR5 expression of Ly6G^hi^Ly6C^lo^, but not Ly6G^lo^Ly6C^hi^, myeloid cells in RACK1-deficient livers was observed by flow cytometry compared to their counterparts in RACK1-sufficient livers (Figure 3E). Therefore, we used an agonistic anti-DR5 antibody. Unexpectedly, flow cytometry analysis indicated that anti-DR5 antibody treatment failed to deplete either Ly6G^hi^Ly6C^lo^ or Ly6G^lo^Ly6C^hi^ myeloid cells in steady-state livers of both* Rack1*^F/F^ and *Rack1*^Δhep^ mice (Figure S1), echoing recent reports of non-apoptotic TRAIL signaling in immune cells 37. Despite that, anti-DR5 antibody treatment showed effects similar to anti-Gr1 antibody treatment for LPS/GalN-driven mortality (Figure 3F) and hepatocyte necrosis (Figure 3G), suggesting that these MDSC functions were blocked. These data confirmed that MDSCs had a protective role in LPS/GalN-induced FH which was weak in wild-type mice. Hepatic RACK1 deficiency resulted in MDSC accumulation in the liver under steady-state conditions and rendered *Rack1*^Δhep^ mice resistant to FH.

### MDSCs inhibit inflammatory cytokine production from macrophages and enhance IKK/NF-κB activation in hepatocytes

**Figure 4:**
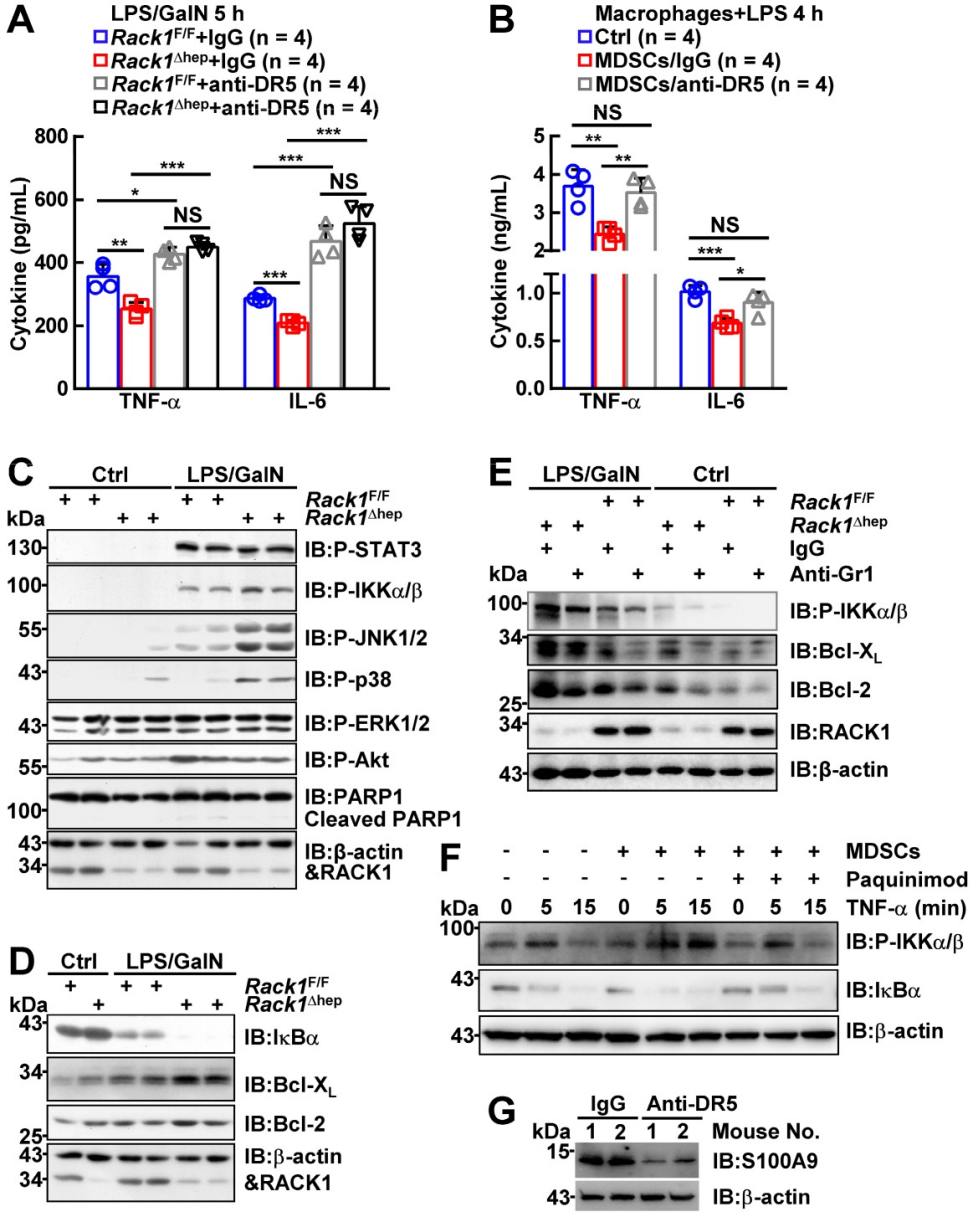
** MDSCs inhibit inflammatory cytokine production from macrophages and enhance IKK/NF-κB activation in hepatocytes.** (**A**) Mice were treated as stated in Figure 3G, inflammatory factors in the liver tissue were measured. (**B**) Bone marrow-derived macrophages from *Rack1*^F/F^ mice were stimulated with 100 ng/mL LPS for 4 h in the presence or absence of the same number of CD11b^+^F4/80^-^Ly6G^hi^Ly6C^lo^ myeloid cells sorted from livers of *Rack1^Δ^*^hep^ mice. TNF-α and IL-6 levels in the supernatants were then determined by ELISA. (**C-E**) After MDSC depletion, as stated in Figure 3A (**E**) or left untreated (**C, D**), 12-week-old *Rack1^Δ^*^hep^ mice and littermate controls were injected with or without LPS/GalN. Livers were isolated 1 h later and subjected to IB analysis with indicated antibodies. P-STAT3, phospho-STAT3; P-IKKα/β, phospho-IKKα/β; P-JNK1/2; phospho-JNK1/2; P-p38, phospho-p38; P-ERK1/2, phospho-ERK1/2; P-Akt, phospho-Akt. (**F**) Primary hepatocytes and Gr1^+^ cells were purified from *Rack1*^F/F^ and *Rack1^Δ^*^hep^ livers, respectively. Gr1^+^ cells were plated above primary hepatocytes in a Transwell in the presence or absence of 50 µg/mL S100A9 inhibitor paquinimod. After overnight incubation, 10 ng/mL murine TNF-α was added to the co-culture system for 0, 5, or 15 min. Then, primary hepatocytes were subjected to IB analysis with indicated antibodies. (**G**) After *Rack1^Δ^*^hep^ mice were injected with the indicated antibody (200 μg/mouse), the liver tissue was subjected to IB analysis 24 h later to examine S100A9 expression. Error bars indicate mean ± SD. The n-value information is shown in the corresponding panel. *p < 0.05; **p < 0.01; ***p < 0.001; NS, not significant.

Next, we explored how MDSC accumulation prevented LPS/GalN-induced liver damage under steady-state conditions. Excessive production of cytokines by macrophages has been demonstrated to play an important role in FH 1. As liver-specific RACK1 deficiency led to reduced levels of TNF-α and IL-6 in this organ 5 h after LPS/GalN administration (Figure 1D), the accumulated MDSCs very likely inhibited inflammatory cytokine production from macrophages as well as T cells. Indeed, anti-DR5 antibody treatment augmented TNF-α and IL-6 levels in both RACK1-sufficient and -deficient livers 5 h after LPS/GalN administration and abrogated the differences between RACK1-sufficient and -deficient livers (Figure 4A). Also, CD11b^+^F4/80^-^Ly6G^hi^Ly6C^lo^ cells sorted from the livers of control antibody-treated, but not anti-DR5 antibody-treated, *Rack1*^Δhep^ mice significantly inhibited TNF-α and IL-6 production from macrophages (Figure 4B). Thus, these accumulated MDSCs exerted protective effects by at least partially inhibiting excessive cytokine production. These data also confirmed the notion that anti-DR5 antibody treatment could block the function of these MDSCs in steady-state livers, although it failed to deplete these cells.

We also examined the signaling pathways of inflammatory factors in *Rack1*^F/F^ and *Rack1*^Δhep^ livers. IB analysis revealed that PARP1 cleavage, an indicator of apoptosis, occurred in *Rack1*^F/F^ livers 1 h after LPS/GalN administration (Figure 4C). Consistent with the protective role of liver-specific RACK1 deficiency, PARP1 cleavage was reduced in *Rack1*^Δhep^ livers (Figure 4C). Furthermore, the phosphorylation of IKKα/β, JNK1/2, and p38, but not STAT3, ERK1/2, and Akt, was enhanced in *Rack1*^Δhep^ livers compared to *Rack1*^F/F^ livers (Figure 4C). Because the IKK/NF-κB pathway determines the degree of liver injury 38, 39, we attempted to further clarify its involvement. Indeed, *Rack1*^Δhep^ livers exhibited more dramatic degradation of IκBα 1 h after LPS/GalN administration than *Rack*1^F/F^ livers (Figure 4D). In addition, the levels of anti-apoptotic proteins Bcl-2 and Bcl-X_L_, known NF-κB target genes 40, in *Rack1*^Δhep^ livers were significantly higher than in *Rack*1^F/F^ livers either under steady-state conditions or after LPS/GalN administration (Figure 4D). Consistent with the above results, deletion of Gr1^+^CD11b^+^ myeloid cells remarkably reversed the augmented IKKα/β phosphorylation and Bcl-2/Bcl-X_L_ expression both under steady-state conditions and after LPS/GalN administration (Figure 4E).

Since the infiltrated MDSCs are S100A9^hi^ (Figure 2D), which has been reported to induce NF-κB activation 41, 42, Gr1^+^ myeloid cells purified from *Rack1*^Δhep^ livers were plated above primary hepatocytes isolated from *Rack*1^F/F^ mice in the Transwell co-culture system. As expected, overnight co-culturing enhanced TNF-α-induced IKKα/β phosphorylation and IκBα degradation in primary hepatocytes (Figure 4F). The S100A9 inhibitor paquinimod significantly reversed augmented IKKα/β phosphorylation and IκBα degradation in the presence of MDSCs (Figure 4F). Thus, the accumulation of MDSCs under steady-state conditions prevented LPS/GalN-induced liver damage through, at least partially, S100A9-mediated enhancement of IKK/NF-κB activity in hepatocytes. Also, anti-DR5 antibody administration led to reduced levels of S100A9 in *Rack1*^Δhep^ livers under steady-state conditions (Figure 4G), suggesting that such treatment could inhibit S100A9 expression in these MDSCs and thereby suppress their ability to enhance IKK/NF-κB activation in hepatocytes.

### RACK1 maintains HDAC1 level in hepatocytes

**Figure 5:**
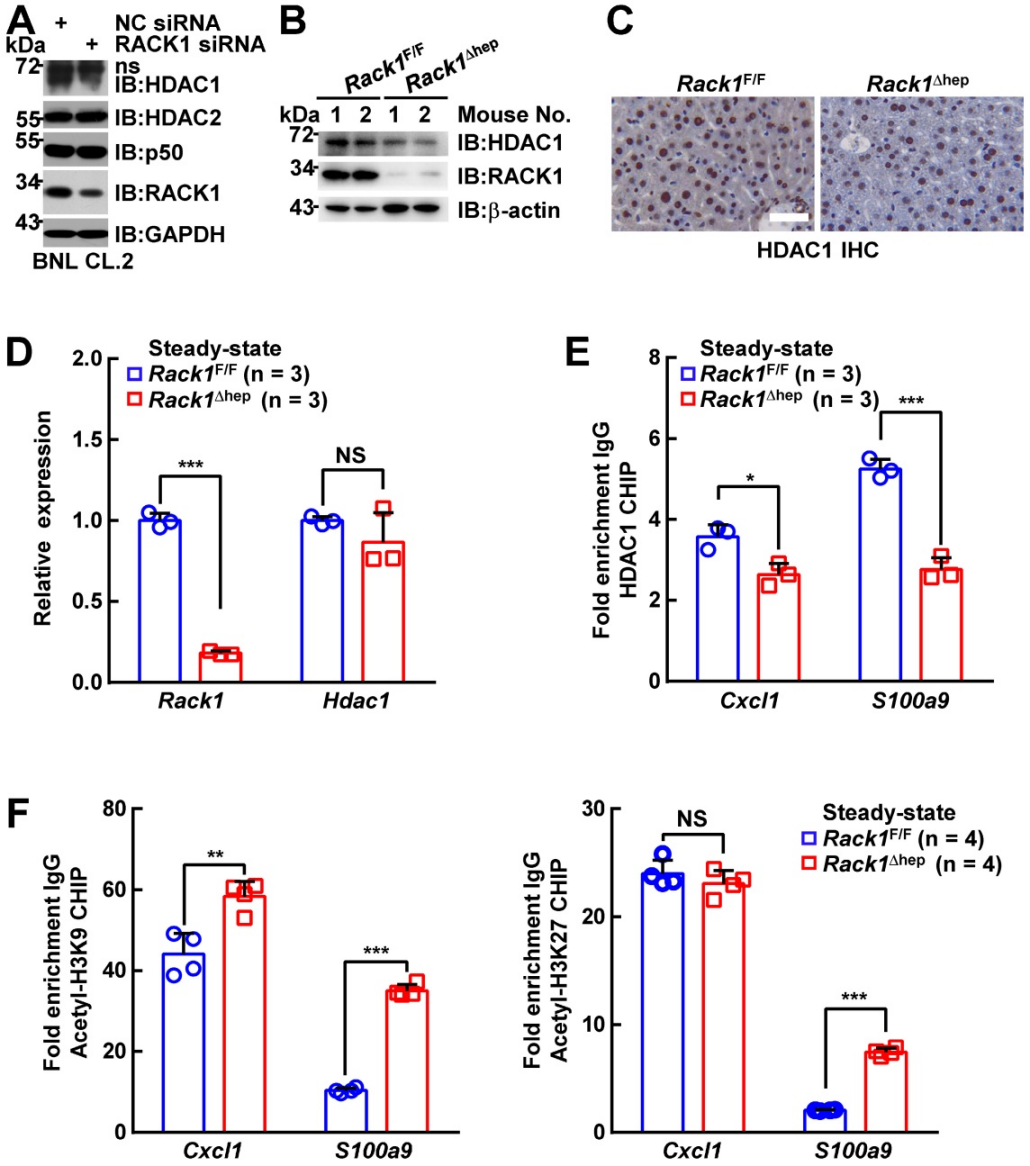
** RACK1 maintains co-repressor HDAC1 protein level in hepatocytes.** (**A**) BNL CL.2 cells were transfected with the indicated siRNA. Whole-cell lysates were harvested 48 h later and subjected to IB analysis with indicated antibodies. ns, non-specific band. (**B-D**) Livers of 12-week-old *Rack1^Δ^*^hep^ mice and littermate controls were subjected to IB (**B**), IHC (**C**, scale bar, 50 µm), and quantitative RT-PCR (**D**) analysis to examine the expression of HDAC1. (**E, F**) ChIP assays of HDAC1 recruitment (**E**) and histone H3K9 and H3K27 acetylation (**F**) at the *Cxcl1* and *S100a9* promoters in livers of 12-week-old *Rack1*^Δhep^ mice and littermate controls. Error bars indicate mean ± SD. The n-value information is shown in the corresponding panel. *p < 0.05; **p < 0.01; ***p < 0.001; NS, not significant.

It has been reported that the *Cxcl1* and *S100a9* promoters are repressed by p50:p50 homodimers complexed with HDAC1 30. We have disclosed that RACK1 regulates the ubiquitylation and stability of HDAC1 and HDAC2 in the developing cerebellum 43. Herein, we examined whether RACK1 maintained HDAC1 and HDAC2 protein levels in hepatocytes. We found that silencing endogenous RACK1 expression by RACK1 siRNA in BNL CL.2 cells led to reduced HDAC1, but not HDAC2 and p50 (Figure 5A). Maintenance of the HDAC1 protein level by RACK1 was further confirmed in *Rack1*^Δhep^ livers by IB (Figure 5B) or IHC (Figure 5C), although livers from *Rack1*^Δhep^ mice exhibited mRNA levels of *Hdac1* comparable to littermate controls (Figure 5D). We used the ChIP assay to confirm HDAC1 recruitment to the *Cxcl1* and *S100a9* promoters, which was diminished in the absence of RACK1 (Figure 5E). Previous reports suggested that HDAC1 induces deacetylation of histone H3K9 and H3K27 at various promotes, thereby repressing promoter activities 44, 45. We also found that histone H3K9 acetylation at the *Cxcl1* and *S100a9* promoters was augmented in *Rack1*^Δhep^ livers compared with *Rack1*^F/F^ livers (Figure 5F). Also, histone H3K27 acetylation at the *S100a9* promoter was elevated in *Rack1*^Δhep^ livers, although it remained unchanged at the *Cxcl1* promoter (Figure 5F).

### RACK1 maintains HDAC1 protein level by direct binding in hepatocytes

**Figure 6:**
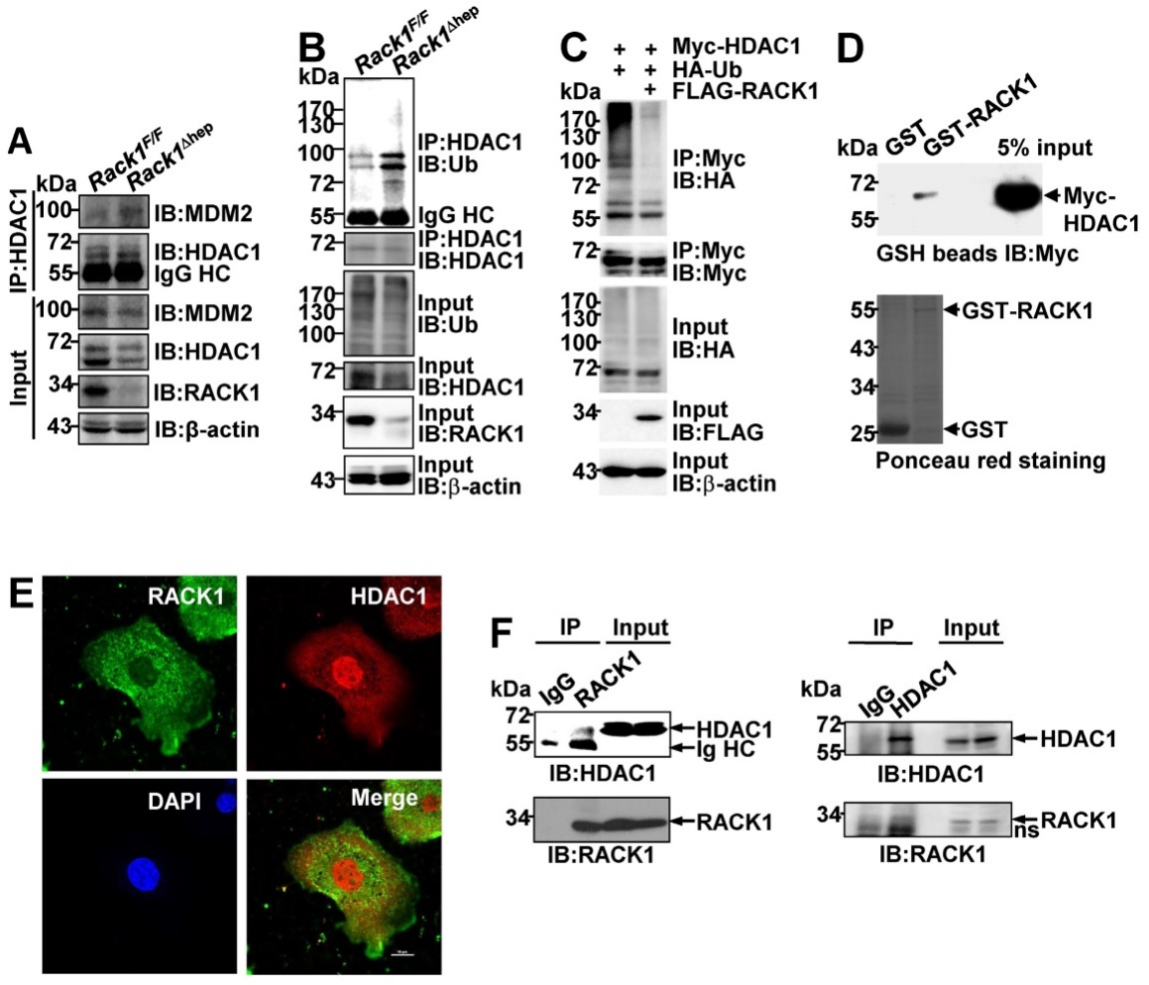
** RACK1 maintains HDAC1 protein level by direct binding in hepatocytes.** (**A**) IB analysis of the interaction between endogenous HDAC1 and MDM2 in *Rack1*^F/F^ and *Rack1*^Δhep^ livers after IP with an anti-HDAC1 antibody. IgG HC, IgG heavy chain. (**B**) Ubiquitination of endogenous HDAC1 in *Rack1*^F/F^ and *Rack1*^Δhep^ livers was analyzed by IB after IP with an anti-HDAC1 antibody. (**C**) BNL CL.2 cells were transfected with mammalian expression vectors as indicated. Ubiquitination of Myc-HDAC1 upon RACK1 over-expression was analyzed by IB after IP with an anti-Myc antibody. Cells were treated with 20 µM MG132 for 6 h, and cell lysates were harvested. WCL, whole-cell lysates. (**D**) GST-RACK1 or GST bound to GSH beads were incubated with lysates of BNL CL.2 cells expressing Myc-HDAC1. Precipitates were subjected to IB. (**E**) Primary hepatocytes were subjected to indirect immunofluorescence analysis with antibodies against RACK1 and HDAC1, then counterstained with DAPI followed by confocal microscopy (scale bar: 10 µm). (**F**) IB analysis of the interaction between endogenous RACK1 and HDAC1 in the liver after IP with an anti-RACK1 antibody (*left*, control antibody: rabbit IgG) or an anti-HDAC1 antibody (*right*, control antibody: rabbit IgG). ns, non-specific band.

It remains unknown how RACK1 controls the HDAC1 protein level. Our previous study indicated that the interaction between HDAC1 and its E3 ligase MDM2 in the developing cerebellum was strongly increased in the absence of RACK1 43. Herein, Co-IP analysis confirmed increased MDM2 binding to HDAC1 in *Rack1*^Δhep^ livers (Figure 6A). Under partially denaturing conditions, IP revealed that liver-specific RACK1 deficiency resulted in enhanced ubiquitination of HDAC1 (Figure 6B), whereas ectopic RACK1 expression in BNL CL.2 cells led to reduced ubiquitination of HDAC1 (Figure 6C). As RACK1 is a multi-functional scaffold protein, we tested whether RACK1 directly interacted with HDAC1 by performing *in vitro* GST pull-down assay. We found substantial exogenous Myc-HDAC1 in lysates of BNL CL.2 cells precipitated specifically by GST-RACK1 but not by GST alone (Figure 6D). We also examined the possible co-localization of RACK1 and HDAC1. Indirect immunofluorescence analysis revealed that RACK1 was predominantly cytoplasmic with a considerable portion distributed in the nucleus, whereas HDAC1 was predominantly nuclear with a substantial fraction in the cytoplasm of primary hepatocytes (Figure 6E). RACK1 and HDAC1 showed partial co-localization in the cytoplasm and nucleus (Figure 6E). The physiological interaction of RACK1 with HDAC1 was confirmed by Co-IP analysis. HDAC1 was present in immuno-precipitates obtained from liver lysates with an antibody against RACK1, whereas no HDAC1 coprecipitated with a control antibody (Figure 6F). Moreover, endogenous RACK1 in the liver coprecipitated with endogenous HDAC1 (Figure 6F). Collectively, our data suggested that RACK1 maintained the HDAC1 protein level by direct binding in hepatocytes.

### Ectopic HDAC1 expression partially reverses the effects of RACK1 deficiency

**Figure 7:**
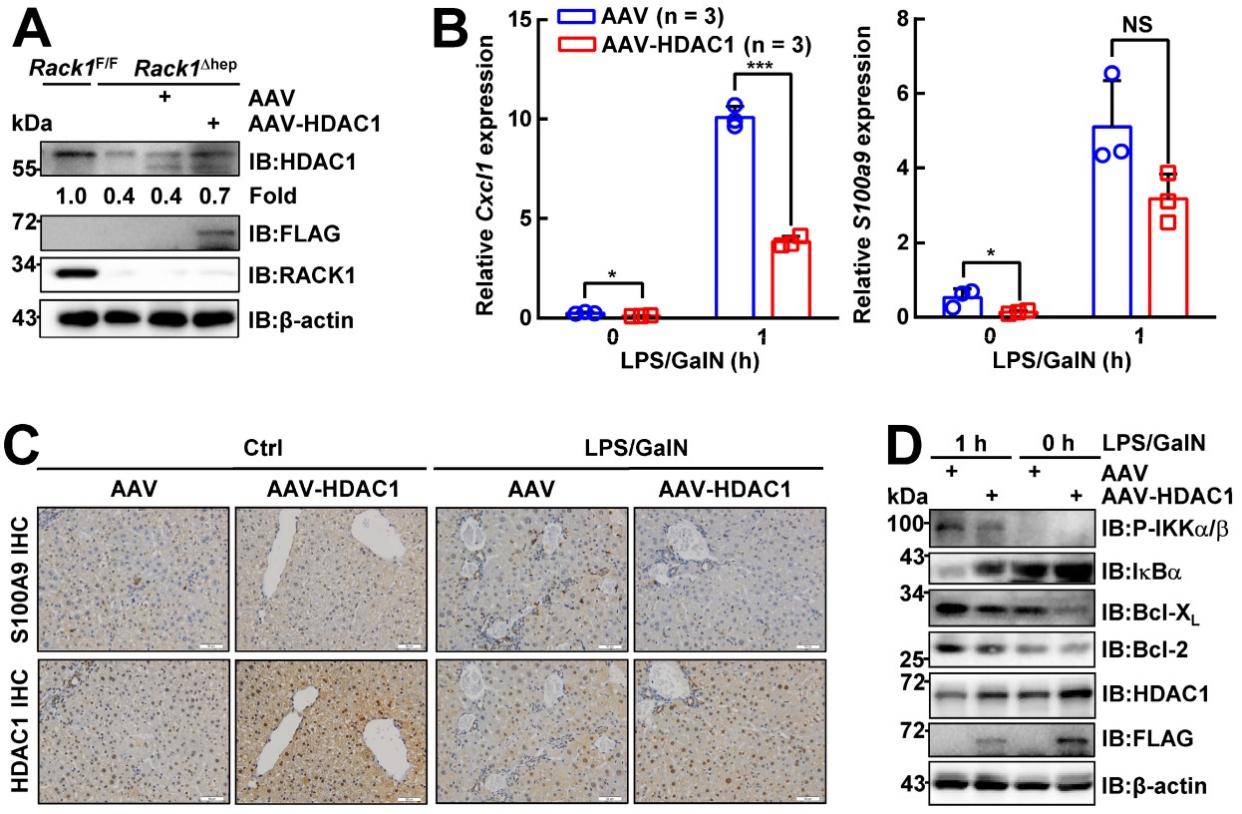
** Ectopic HDAC1 expression partially reverses the effects of hepatic RACK1 deficiency.** 8-week-old *Rack1*^Δhep^ mice were randomly divided into two groups. The control group was injected with 5 × 10^11^ v.g. AAV8-CMV-GFP in 200 µL PBS via the caudal vein, while the other group was injected with the same dose of AAV8-expressing murine FLAG-tagged HDAC1 (AAV8-HDAC1). Four weeks after injection, mice were used for the following experiments. (**A**) IB analysis of exogenous HDAC1 expression. (**B-D**) Quantitative RT-PCR analysis of *Cxcl1* and *S100a9* expression (**B**), IHC analysis of S100A9 and HDAC1 (**C**, scale bar, 50 µm), or IB analysis of the activation of the IKK/NF-κB pathway (**D**) in the liver tissue before and 1 h after LPS/GalN injection. Error bars indicate mean ± SD. The n-value information is shown in the corresponding panel. *p < 0.05; **p < 0.01; ***p < 0.001; NS, not significant.

To confirm whether reduced HDAC1 protein level played a key role in the protective effects of hepatic RACK1 deficiency against FH, AAV8 expressing murine FLAG-tagged HDAC1 was injected into the tail vein of 8-week-old *Rack1*^Δhep^ mice. AAV8 preferentially infects hepatocytes, and transgene expression persists for over 11 months 46, 47. Four weeks after the injection, IB analysis confirmed exogenous HDAC1 expression, partially reversing the reduced protein level of HDAC1 in *Rack1*^Δhep^ livers (Figure 7A). Quantitative RT-PCR revealed that the basal levels of *Cxcl1* and *S100a9* expression decreased in AAV8-HDAC1-transduced *Rack1*^Δhep^ livers compared to AAV8-vector-transduced counterparts (Figure 7B). After LPS/GalN challenge, the upregulation of *Cxcl1* was suppressed by AAV8-HDAC1 transduction. A similar trend was also observed with *S100a9,* although it failed to reach statistical significance (*p* = 0.0761) (Figure 7B). IHC revealed that the HDAC1 protein level was heterogenous in AAV8-HDAC1-transduced *Rack1*^Δhep^ livers. In certain areas with upregulated HDAC1, accumulation of S100A9^hi^ cells before and after LPS/GalN challenge was alleviated (Figure 7C). As for the signaling, ectopic HDAC1 expression inhibited LPS/GalN-triggered IKKα/β phosphorylation and IκBα degradation in *Rack1*^Δhep^ livers (Figure 7D). Moreover, Bcl-2 and Bcl-X_L_ levels were suppressed by AAV8-HDAC1 transduction under steady-state conditions and after LPS/GalN administration (Figure 7D).

### RACK1 is downregulated during the induction of FH

**Figure 8:**
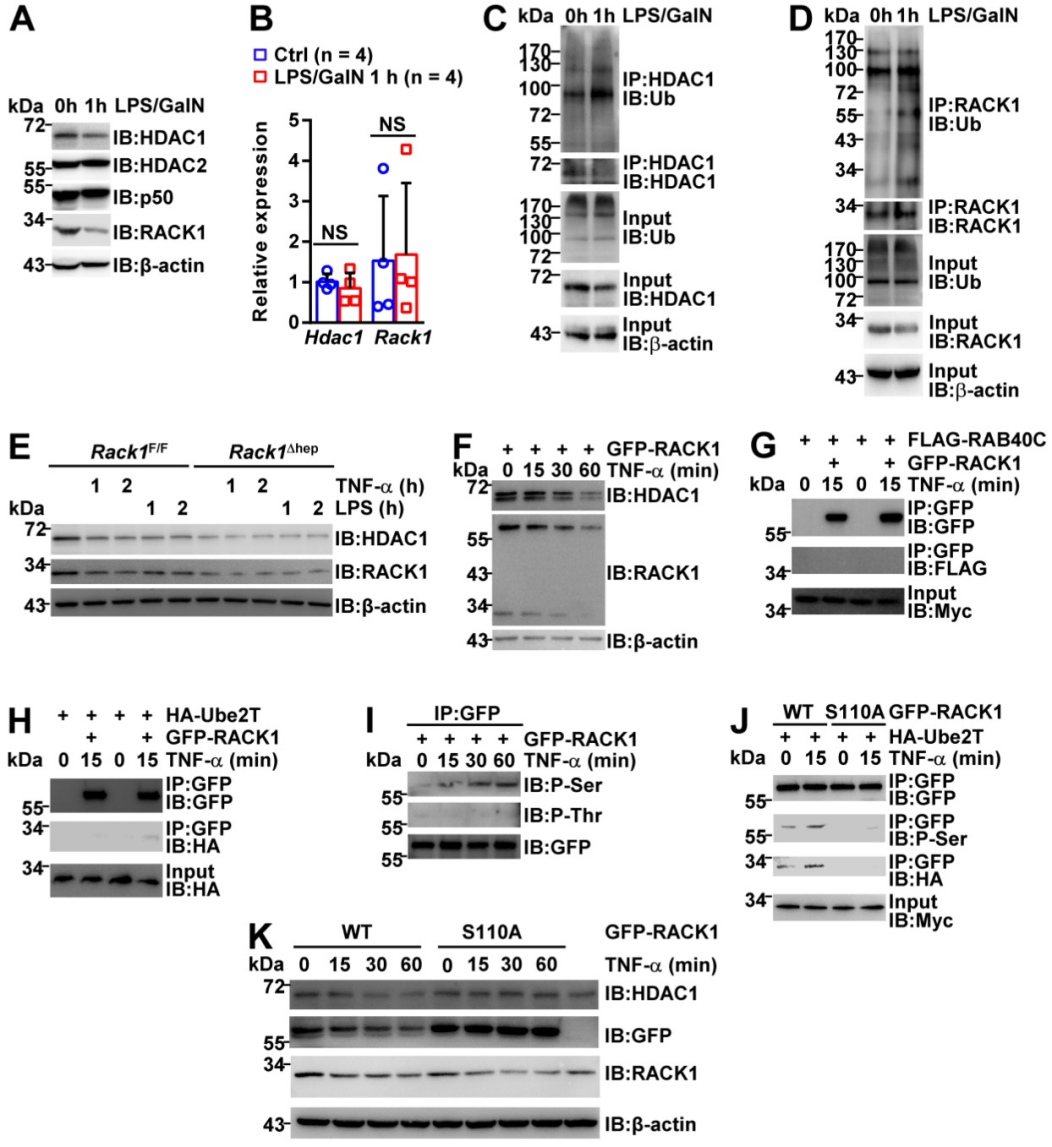
** RACK1 is downregulated during FH induction.** (**A-D**) 12-week-old wild-type mice were injected with or without LPS/GalN. Primary hepatocytes were purified 1 h later and subjected to IB (**A**), quantitative RT-PCR (**B**), and *in vivo* ubiquitination assay with IP (**C, D**). (**E**) Primary hepatocytes were purified from 12-week-old *Rack1*^Δhep^ mice and littermate controls. After stimulation with 10 ng/mL murine TNF-α or 100 ng/mL LPS for 0, 1, or 2 h, primary hepatocytes were subjected to IB analysis with indicated antibodies. (**F-K**) BNL CL.2 cells were transfected with mammalian expression vectors as indicated. After 24 h, cells were stimulated with 10 ng/mL murine TNF-α for indicated time periods. Whole-cell lysates were then prepared in IP lysis buffer and subjected to IB analysis directly (**F**, **K**) or after IP with an antibody against GFP (**G-J**). P-Ser, phospho-serine; P-Thr, phospho-threonine; WT, wild-type. Error bars indicate mean ± SD. The n-value information is shown in the corresponding panel. *p < 0.05; **p < 0.01; ***p < 0.001; NS, not significant.

The HDAC1 protein level in either AAV8-vector or AAV8-HDAC1-transduced* Rack1*^Δhep^ livers was reduced after LPS/GalN challenge (Figure 7C and 7D), suggesting that HDAC1 expression was actively downregulated during the induction of FH. As RACK1 protein level remained unchanged in the liver tissue (Figure 4A-C), we speculated that HDAC1 downregulation could be due to immune cell infiltration. Therefore, we purified primary hepatocytes from wild-type mice before and after LPS/GalN challenge. IB analysis revealed reduced protein levels of HDAC1 and RACK1, but not HDAC2 and p50, after LPS/GalN challenge (Figure 8A). Rapid reduction of HDAC1 and RACK1 during FH induction was suggestive of ubiquitin-proteasome-dependent degradation. Recent studies suggested that RACK1 might also undergo ubiquitination under specific circumstances 48-50. Our quantitative RT-PCR data also indicated that *Hdac1* and *Rack1* mRNA levels were unchanged under the same conditions (Figure 8B). Furthermore, under partially denaturing conditions, IP demonstrated enhanced ubiquitination of HDAC1 (Figure 8C) and RACK1 (Figure 8D) after LPS/GalN challenge. To study the regulatory relationship between HDAC1 and RACK1 downregulation, primary hepatocytes were purified from *Rack1*^F/F^ and *Rack1*^Δhep^ mice and stimulated with TNF-α or LPS for 0, 1, or 2 h. IB analysis showed that inflammatory stimulation led to reduced protein levels of both HDAC1 and RACK1 in primary hepatocytes from *Rack1*^F/F^ but not from *Rack1*^F/F^ mice (Figure 8E). To clarify the time course of HDAC1 and RACK1 downregulation, BNL CL.2 cells expressing GFP-RACK1 were stimulated with TNF-α for 0, 15, 30, and 60 min. IB analysis indicated that the downregulation of both exogenous and endogenous RACK1 preceded that of HDAC1 and started at 15 min after TNF-α treatment (Figure 8F). Therefore, the downregulation of HDAC1 during FH induction resulted, at least partially, from the reduction of RACK1.

Next, explored the mechanism(s) underlying RACK1 ubiquitination and degradation during FH induction. A previous study reported that an atypical Rab protein and small GTPase RAB40C functions as a putative ubiquitin E3 ligase for RACK1 in HEK-293 cells 48. Another group demonstrated that an ubiquitin-conjugating enzyme Ube2T directly interacts with RACK1 and induces RACK1 ubiquitination and degradation independent of the E3 ligase in gastric cancer cells 49. We failed to detect the interaction between RAB40C and RACK1 in BNL CL.2 cells before and after TNF-α treatment for 15 min (Figure 8G). By contrast, the barely detectable interaction between Ube2T and RACK1 in BNL CL.2 cells was augmented after TNF-α treatment for 15 min (Figure 8H), explaining the enhanced ubiquitination of RACK1 during FH induction (Figure 8D).

Because our previous studies indicated that RACK1 phosphorylation could actively modulate its adaptor function 19, 51, we speculated that RACK1 might undergo phosphorylation after inflammatory stimulation, enhancing its binding to Ube2T. For this purpose, GFP-RACK1 was immunoprecipitated from BNL CL.2 cells before and after TNF-α treatment. IB analysis revealed that TNF-α stimulated RACK1 serine phosphorylation, but not threonine phosphorylation, in BNL CL.2 cells (Figure 8I). Based on our previous study showing RACK1 phosphorylation at serine 110 51, we used site-directed mutagenesis to replace serine 110 with non-phosphorylatable alanine (S110A). This S100A mutation blocked the basal and TNF-α-induced serine phosphorylation of RACK1 and inhibited the interaction between RACK1 and Ube2T (Figure 8J). Intriguingly, S100A mutant remained stable under the conditions that endogenous RACK1 and exogenous wild-type protein rapidly underwent TNF-α-induced degradation (Figure 8K). Furthermore, overexpression of S110A mutant blocked TNF-α-induced degradation of endogenous HDAC1 (Figure 8K). Thus, inflammatory stimulation triggered RACK1 phosphorylation at serine 110, enhancing its binding to Ube2T and promoting its ubiquitination and degradation.

## Discussion

RACK1 promotes autophagy and protects against TNF-α-induced cell death, suggesting that *in vivo* RACK1 loss-of-function aggravates FH. By contrast, we observed that liver-specific RACK1 deficiency rendered mice resistant to FH. Further exploration revealed that by directly binding to HDAC1, RACK1 maintained the HDAC1 protein level in hepatocytes. HDAC1 is known to bind to the *Cxcl1* and *S100a9* promoters and represses promoter activities by inducing deacetylation of histone H3K9 and H3K27 44, 45. In the absence of hepatic RACK1, however, the repression by HDAC1 is lost. Chemokines are expressed by hepatocytes, followed by MDSC recruitment into the liver under steady-state conditions. MDSCs are characterized by their ability to inhibit T cells 4. In this study, we have shown that these myeloid cells exerted protective effects through at least two novel mechanisms: inhibition of inflammatory cytokine production from macrophages and contribution to the activation of the IKK/NF-κB pathway in hepatocytes. Consequently, liver injury is prevented. S100A9 is a key mediator for these MDSCs to enhance IKK/NF-κB activation in hepatocytes. Nevertheless, how these accumulated MDSCs suppress macrophages to secret inflammatory cytokines remains unknown. Since microarray-based transcriptome analysis revealed comparable levels of *Tnf*, *Il6*, and *Il1b* in *Rack1*^F/F^ and *Rack1*^Δhep^ livers before and after LPS/GalN challenge, the inhibition might occur at the post-transcriptional level. Future studies are required to address this issue.

Intriguingly, MDSCs failed to directly activate IKKα/β in co-cultured hepatocytes *in vitro*, although they facilitated IKKα/β activation by pro-inflammatory stimulus TNF-α (Figure 4D). However, *in vivo* RACK1 deficiency led to higher levels of IKKα/β phosphorylation and Bcl-2/Bcl-X_L_ expression in the liver under steady-state conditions that could be reversed after MDSC depletion (Figure 4C and 4D). Gradients from gut microbiota may trigger weak TNF-α production from Kupffer cells under steady-state conditions synergizing with MDSCs to enhance the activity of the IKK/NF-κB pathway.

Even though RACK1 regulates the ubiquitylation and stability of HDAC1 and HDAC2 in the developing cerebellum 43, RACK1 did not affect the HDAC2 protein level in hepatocytes (Figure 5A and 8A). The regulation of HDAC2 stability by RACK1 may require specific component(s), which are deficient in hepatocytes. As for HDAC1, its ectopic expression mediated by AAV8 in RACK1-deficient hepatocytes was not very efficient (Figure 7A) and failed to reverse the resistance of *Rack1*^Δhep^ mice to FH, probably because exogenous HDAC1 was not stable in the absence of RACK1. Ectopic expression of a ubiquitination-defective mutant of HDAC1 might be more efficient. It is also possible that other mechanism(s) contribute to the recruitment of MDSCs into *Rack1*^Δhep^ livers.

RACK1 protein level was rapidly downregulated in wild-type mice after FH induction associated with reduced HDAC1 (Figure 8A). Downregulation of HDAC1 could occur in RACK1-deficient livers during FH induction (Figure 7C and 7D) but not in RACK1-deficient primary hepatocytes after stimulation with TNF-α or LPS (Figure 8E). Since the liver microenvironment is very complicated, especially after FH induction, HDAC1 reduction in RACK1-deficient livers might occur in various RACK1-sufficient cells and/or other factor(s) in the microenvironment induce HDAC1 downregulation in the absence of RACK1. A confirmation of these possibilities awaits future studies. Nevertheless, our data indicated that HDAC1 downregulation during FH induction resulted, at least partially, from the reduction of RACK1. Decreased HDAC1 is expected to contribute to MDSC recruitment after FH induction because ectopic HDAC1 expression inhibited chemokine upregulation after LPS/GalN challenge (Figure 7B). However, this regulation was relatively weak since targeting MDSCs in wild-type mice only marginally affected the mortality (Figure 3B and 3F), liver damage (Figure 3C, 3D, and 3G), and inflammation (Figure 4A). Augmentation of this process might contribute to the treatment of FH. Thus, it is necessary to clarify the molecular mechanism(s) underlying the rapid reduction of RACK1. Herein, we have shown that inflammatory stimulation triggered RACK1 phosphorylation at serine 110, enhancing its binding to Ube2T and thereby promoting its ubiquitination and degradation. Future studies should be directed to identify the corresponding kinase(s) and phosphatase(s) because targeting these components might facilitate the interference of FH in the clinic.

## Supplementary Material

Supplementary figure.

## Abbreviations

ALT: alanine aminotransferase
AST: aspartate transaminase
ChIP: chromatin immunoprecipitation
Con A: concanavalin A
ERK: extracellular signal-regulated kinase
GSH: glutathione-Sepharose
GST: glutathione S-transferase
CXCL1: chemokine (C-X-C motif) ligand 1
FH: fulminant hepatitis
GalN: D-Galactosamine
HDAC1: histone deacetylase 1
IB: immunoblotting
IHC: immunohistochemistry
IKK: IκB kinase
IP: immunoprecipitation
JNK: c-Jun N-terminal kinase
LPS: lipopolysaccharide
MDM2: mouse double minute 2
MDSCs: myeloid-derived suppressor cells
MKK7: mitogen-activated protein kinase kinase 7
PKC: protein kinase C
RACK1: receptor for activated C kinase 1
siRNA: small interfering RNA
STAT3: signal transducer and activator of transcription 3
S100A9: S100 calcium-binding protein A9
TNF-α: tumor necrosis factor alpha

## References

[1] Xia Y, Wang P, Yan N, Gonzalez FJ, Yan T (2021). Withaferin A alleviates fulminant hepatitis by targeting macrophages and NLRP3. Cell Death Dis.

[2] Diaz-Buxo JA, Blumenthal S, Hayes D, Gores P, Gordon B (1997). Galactosamine-induced fulminant hepatic necrosis in unanesthetized canines. Hepatology.

[3] Gantner F, Leist M, Lohse AW, Germann PG, Tiegs G (1995). Concanavalin A-induced T-cell-mediated hepatic injury in mice: the role of tumor necrosis factor. Hepatology.

[4] Bronte V, Brandau S, Chen SH, Colombo MP, Frey AB, Greten TF (2016). Recommendations for myeloid-derived suppressor cell nomenclature and characterization standards. Nat Common.

[5] Sarra M, Cupi ML, Bernardini R, Ronchetti G, Monteleone I, Ranalli M (2013). IL-25 prevents and cures fulminant hepatitis through a myeloid-derived suppressor cell-dependent mechanism. Hepatology.

[6] Diao W, Jin F, Wang B, Zhang CY, Chen J, Zen K (2014). The protective role of myeloid-derived suppressor cells in concanavalin A-induced hepatic injury. Protein Cell.

[7] Wu D, Shi Y, Wang C, Chen H, Liu Q, Liu J (2017). Activated NKT cells facilitated functional switch of myeloid-derived suppressor cells at inflammation site in fulminant hepatitis mice. Immunobiology.

[8] Wang H, Li X, Dong G, Yan F, Zhang J, Shi H (2021). Toll-like receptor 4 inhibitor TAK-242 improves fulminant hepatitis by regulating accumulation of myeloid-derived suppressor cell. Inflammation.

[9] Ron D, Chen CH, Caldwell J, Jamieson L, Orr E, Mochly-Rosen D (1994). Cloning of an intracellular receptor for protein kinase C: a homolog of the beta subunit of G proteins. Proc Natl Acad Sci USA.

[10] Bourd-Boittin K, Le Pabic H, Bonnier D, L'Helgoualc'h A, Theret N (2008). RACK1, a new ADAM12 interacting protein. Contributing to liver fibrogenesis. J Biol Chem.

[11] Jia D, Duan F, Peng P, Sun L, Liu X, Wang L (2013). Up-regulation of RACK1 by TGF-β1 promotes hepatic fibrosis in mice. PLoS One.

[12] Liu M, Peng O, Wang J, Wang L, Duan F, Jia D (2015). RACK1-mediated translation control promotes liver fibrogenesis. Biochem Biophys Res Commun.

[13] Guo Y, Wang W, Wang J, Feng J, Wang Q, Jin J (2013). Receptor for activated C kinase 1 promotes hepatocellular carcinoma growth by enhancing mitogen-activated protein kinase kinase 7 activity. Hepatology.

[14] Ruan Y, Sun L, Hao Y, Wang L, Xu J, Zhang W (2012). Ribosomal RACK1 promotes chemoresistance and growth in human hepatocellular carcinoma. J Clin Invest.

[15] Majzoub K, Hafirassou ML, Meignin C, Goto A, Marzi S, Fedorova A (2014). RACK1 controls IRES-mediated translation of viruses. Cell.

[16] Subramani C, Nair VP, Anang S, Das Mandal S, Pareek M, Kaushik N (2018). Host-virus protein interaction network reveals the involvement of multiple host processes in the life cycle of hepatitis E virus. mSystems.

[17] Jia B, Guo M, Li G, Yu D, Zhang X, Lan K (2015). Hepatitis B virus core protein sensitizes hepatocytes to tumor necrosis factor-induced apoptosis by suppression of the phosphorylation of mitogen-activated protein kinase kinase 7. J Virol.

[18] Wang Q, Zhou S, Wang JY, Cao J, Zhang X, Wang J (2015). RACK1 antagonizes TNF-α-induced cell death by promoting p38 activation. Sci Reports.

[19] Zhao Y, Wang Q, Qiu G, Zhou S, Jing Z, Wang J (2015). RACK1 promotes autophagy by enhancing the Atg14L-Beclin 1-Vps34-Vps15 complex formation upon phosphorylation by AMPK. Cell Rep.

[20] Su F, Schneider RJ (1997). Hepatitis B virus HBx protein sensitizes cells to apoptotic killing by tumor necrosis factor alpha. Proc Natl Acad Sci USA.

[21] Lehmann V, Freudenberg MA, Galanos C (1987). Lethal toxicity of lipopolysaccharide and tumor necrosis factor in normal and D-galactosamine-treated mice. J Exp Med.

[22] Hishinuma I, Nagakawa J, Hirota K, Miyamoto K, Tsukidate K, Yamanaka T (1990). Involvement of tumor necrosis factor-alpha in development of hepatic injury in galactosamine-sensitized mice. Hepatology.

[23] Amir M, Zhao E, Fontana L, Rosenberg H, Tanaka K, Gao G (2013). Inhibition of hepatocyte autophagy increases tumor necrosis factor-dependent liver injury by promoting caspase-8 activation. Cell Death Differ.

[24] Klaunig JE, Goldblatt PJ, Hinton DE, Lipsky MM, Chacko J, Trump BF (1981). Mouse liver cell culture. I. Hepatocyte isolation. *In vitro*.

[25] Livak KJ, Schmittgen TD (2001). Analysis of relative gene expression data using real time quantitative PCR and the 2^-ΔΔ*C*T^ method. Methods.

[26] Schmittgen TD, Livak KJ (2008). Analyzing real-time PCR data by the comparative C_T_ method. Nat Protoc.

[27] Xie P, Zhang M, He S, Lu K, Chen Y, Xing G (2014). The covalent modifier Nedd8 is critical for the activation of Smurf1 ubiquitin ligase in tumorigenesis. Nat Commun.

[28] Li YM, Liu ZY, Wang JC, Yu JM, Li ZC, Yang HJ (2019). Receptor-interacting protein kinase 3 deficiency recruits myeloid-derived suppressor cells to hepatocellular carcinoma through the chemokine (C-X-C motif) ligand 1-chemokine (C-X-C motif) receptor 2 axis. Hepatology.

[29] Cheng P, Corzo CA, Luetteke N, Yu B, Nagaraj S, Bui MM (2008). Inhibition of dendritic cell differentiation and accumulation of myeloid-derived suppressor cells in cancer is regulated by S100A9 protein. J Exp Med.

[30] Wilson CL, Jurk D, Fullard N, Banks P, Page A, Luli S (2015). NFκB1 is a suppressor of neutrophil-driven hepatocellular carcinoma. Nat Commun.

[31] Feng PH, Lee KY, Chang YL, Chan YF, Kuo LW, Lin TY (2012). CD14(+)S100A9(+) monocytic myeloid-derived suppressor cells and their clinical relevance in non-small cell lung cancer. Am J Respir Crit Care Med.

[32] Wang Y, Yin K, Tian J, Xia X, Ma J, Tang X (2019). Granulocytic myeloid-derived suppressor cells promote the stemness of colorectal cancer cells through exosomal S100A9. Adv Sci.

[33] Boivin G, Faget J, Ancey PB, Gkasti A, Mussard J, Engblom C (2020). Durable and controlled depletion of neutrophils in mice. Nat Commun.

[34] Veglia F, Sanseviero E, Gabrilovich DI (2021). Myeloid-derived suppressor cells in the era of increasing myeloid cell diversity. Nat Rev Immunol.

[35] Chen J, Sun HW, Yang YY, Chen HT, Yu XJ, Wu WC (2021). Reprogramming immunosuppressive myeloid cells by activated T cells promotes the response to anti-PD-1 therapy in colorectal cancer. Signal Transduct Target Ther.

[36] He YM, Li X, Perego M, Nefedova Y, Kossenkov AV, Jensen EA (2018). Transitory presence of myeloid-derived suppressor cells in neonates is critical for control of inflammation. Nat Med.

[37] Alves LC, Corazza N, Micheau O, Krebs P (2021). The multifaceted role of TRAIL signaling in cancer and immunity. FEBS J.

[38] Beraza N, Ludde T, Assmus U, Roskams T, Borght SV, Trautwein C (2007). Hepatocyte-specific IKK gamma/NEMO expression determines the degree of liver injury. Gastroenterology.

[39] Geisler F, Algul H, Paxian S, Schmid RM (2007). Genetic inactivation of RelA/p65 sensitizes adult mouse hepatocytes to TNF-induced apoptosis *in vivo* and *in vitro*. Gastroenterology.

[40] Feuerhake F, Kutok JL, Monti S, Chen W, LaCasce AS, Cattoretti G (2005). NFκB activity, function, and target-gene signatures in primary mediastinal large B-cell lymphoma and diffuse large B-cell lymphoma subtypes. Blood.

[41] Benedyk M, Sopalla C, Nacken W, Bode G, Melkonyan H, Banfi B (2007). HaCat keratinocytes overexpressing the S100 proteins S100A8 and S100A9 show increased NADPH oxidase and NF-kappaB activities. J Invest Dermatol.

[42] Sunahori K, Yamamura M, Yamana J, Takasugi K, Kawashima M, Yamamoto H (2006). The S100A8/A9 heterodimer amplifies proinflammatory cytokine production by macrophages via activation of nuclear factor kappa B and p38 mitogen-activated protein kinase in rheumatoid arthritis. Arthritis Res Ther.

[43] Yang H, Zhu Q, Cheng J, Wu Y, Fan M, Zhang J (2019). Opposite regulation of Wnt/β-catenin and Shh signaling pathways by Rack1 controls mammalian cerebellar development. Proc Natl Acad Sci U S A.

[44] Tencer AH, Cox KL, Di L, Bridgers JB, Lyu J, Wang X (2017). Covalent modification of histone H3K9 promote binding of CHD3. Cell Rep.

[45] Liu Y, Jiang L, Sun C, Ireland N, Shah YM, Liu Y (2018). Insulin/Snail1 axis ameliorates fatty liver disease by epigenetically suppressing lipogenesis. Nat Commun.

[46] Vozenilek AE, Blackburn CMR, Schilke RM, Chandran S, Castore R, Klein RL (2018). AAV8-mediated overexpression of mPCSK9 in liver differs between male and female mice. Atherosclerosis.

[47] McNamara EL, Taylor RL, Clayton JS, Goullee H, Dilworth JS, Pinos T (2020). Systemic AAV8-mediated delivery of a functional copy of muscle glycogen phosphorylase (Pygm) ameliorates disease in a murine model of McArdle disease. Human Mol Genet.

[48] Day JP, Whiteley E, Freeley M, Long A, Malacrida B, Kiely P (2018). RAB40C regulates RACK1 stability via the ubiquitin-proteasome system. Future Sci OA.

[49] Yu Z, Jiang X, Qin L, Deng H, Wang J, Ren W (2021). A novel UBE2T inhibitor suppresses Wnt/β-catenin signaling hyperactivation and gastric cancer progression by blocking RACK1 ubiquitination. Oncogene.

[50] Wu B, Chang N, Xi H, Xiong J, Zhou Y, Wu Y (2021). PHB2 promotes tumorigenesis via RACK1 in non-small cell lung cancer. Theranostics.

[51] Qin C, Niu C, Shen Z, Zhang Y, Liu G, Hou C (2021). RACK1 T50 phosphorylation by AMPK potentiates its binding with IRF3/7 and inhibition of type 1 IFN production. J Immunol.

