# Supplementary material for: Hepatic RACK1 deficiency protects against fulminant hepatitis through myeloid-derived suppressor cells
Source: Theranostics. 2022 Feb 14;12(5):2248–65. doi: 10.7150/thno.65916 (PMC8899575; doi:10.7150/thno.65916)
Supplement: Supplementary file 1 — Supplementary figure. [file thnov12p2248s1.pdf]

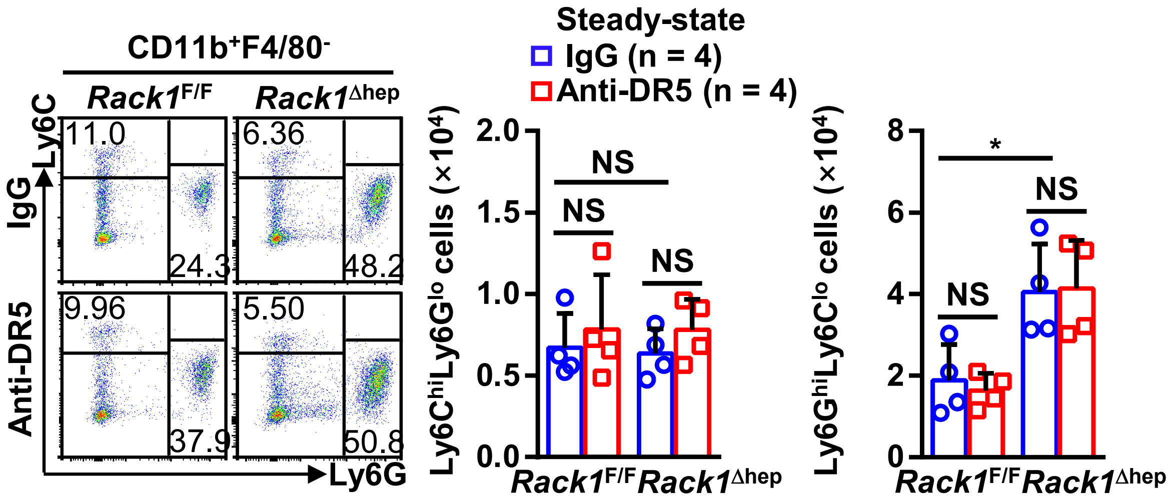

Figure S1. Anti-DR5 antibody treatment can't deplete either Ly6G^{hi}Ly6C^{lo} or Ly6G^{lo}Ly6C^{hi} myeloid cells in steady-state livers.

12-week-old *Rack1*$^{\Delta hep}$ mice and littermate controls were intraperitoneally injected with IgG2b isotype control antibody or anti-mouse DR5 antibody at the dose of 200 μg/mouse. Then liver mononuclear cells were purified 24 h later and subjected to flow cytometry analysis of Ly6G^{hi}Ly6C^{lo} and Ly6G^{lo}Ly6C^{hi} subsets in CD45^{+}CD11b^{+}F4/80^{-} cells Representative plots (*left*) and the statistical data (*right*) are shown. Error bars indicate mean ± SD. The n-value information is shown in the panel. *$p < 0.05$; **$p < 0.01$; ***$p < 0.001$; NS, not significant.
